# AlphaFold2 Modeling and Molecular Dynamics Simulations of the Conformational Ensembles for the SARS-CoV-2 Spike Omicron JN.1, KP.2 and KP.3 Variants: Mutational Profiling of Binding Energetics Reveals Epistatic Drivers of the ACE2 Affinity and Escape Hotspots of Antibody Resistance

**DOI:** 10.3390/v16091458

**Published:** 2024-09-13

**Authors:** Nishank Raisinghani, Mohammed Alshahrani, Grace Gupta, Gennady Verkhivker

**Affiliations:** 1 Keck Center for Science and Engineering, Graduate Program in Computational and Data Sciences, Schmid College of Science and Technology, Chapman University, Orange, CA 92866, USA; rai.r.nick@gmail.com (N.R.); alshahrani@chapman.edu (M.A.); grgupta@chapman.edu (G.G.); 2 Department of Structural Biology, Stanford University, Stanford, CA 94305, USA; 3 Department of Biomedical and Pharmaceutical Sciences, Chapman University School of Pharmacy, Irvine, CA 92618, USA

**Keywords:** SARS-CoV-2 spike protein, Omicron subvariants, ACE2 host receptor, molecular dynamics, protein stability, network analysis, mutational scanning, binding energetics, epistasis, immune escape, monoclonal antibodies, evolutionary mechanisms

## Abstract

The most recent wave of SARS-CoV-2 Omicron variants descending from BA.2 and BA.2.86 exhibited improved viral growth and fitness due to convergent evolution of functional hotspots. These hotspots operate in tandem to optimize both receptor binding for effective infection and immune evasion efficiency, thereby maintaining overall viral fitness. The lack of molecular details on structure, dynamics and binding energetics of the latest FLiRT and FLuQE variants with the ACE2 receptor and antibodies provides a considerable challenge that is explored in this study. We combined AlphaFold2-based atomistic predictions of structures and conformational ensembles of the SARS-CoV-2 spike complexes with the host receptor ACE2 for the most dominant Omicron variants JN.1, KP.1, KP.2 and KP.3 to examine the mechanisms underlying the role of convergent evolution hotspots in balancing ACE2 binding and antibody evasion. Using the ensemble-based mutational scanning of the spike protein residues and computations of binding affinities, we identified binding energy hotspots and characterized the molecular basis underlying epistatic couplings between convergent mutational hotspots. The results suggested the existence of epistatic interactions between convergent mutational sites at L455, F456, Q493 positions that protect and restore ACE2-binding affinity while conferring beneficial immune escape. To examine immune escape mechanisms, we performed structure-based mutational profiling of the spike protein binding with several classes of antibodies that displayed impaired neutralization against BA.2.86, JN.1, KP.2 and KP.3. The results confirmed the experimental data that JN.1, KP.2 and KP.3 harboring the L455S and F456L mutations can significantly impair the neutralizing activity of class 1 monoclonal antibodies, while the epistatic effects mediated by F456L can facilitate the subsequent convergence of Q493E changes to rescue ACE2 binding. Structural and energetic analysis provided a rationale to the experimental results showing that BD55-5840 and BD55-5514 antibodies that bind to different binding epitopes can retain neutralizing efficacy against all examined variants BA.2.86, JN.1, KP.2 and KP.3. The results support the notion that evolution of Omicron variants may favor emergence of lineages with beneficial combinations of mutations involving mediators of epistatic couplings that control balance of high ACE2 affinity and immune evasion.

## 1. Introduction

The spike (S) glycoprotein of SARS-CoV-2 plays a pivotal role in the virus’s ability to enter host cells. The wealth of structural and biochemical investigations conducted on the S glycoprotein have provided crucial insights into the mechanisms that regulate virus transmission and immune evasion. The conformational flexibility of the S glycoprotein, particularly within the S1 subunit and its various domains, which includes the N-terminal domain (NTD), the receptor-binding domain (RBD) and two structurally conserved subdomains—SD1 and SD2—allows it to adapt to different stages of the viral entry process [1,2,3,4,5,6,7,8,9]. The transitions between closed and open states, driven by conformational changes in the NTD and RBD, enable the virus to effectively engage with host cell receptors while evading immune surveillance through structural variability [10,11,12,13,14,15]. Understanding these structural dynamics is crucial for designing therapeutics and vaccines that target the S protein, aiming to disrupt viral entry and prevent infection by SARS-CoV-2. Biophysical investigations have delineated how thermodynamic principles and kinetic factors govern the mechanisms of the S protein [16,17,18]. These studies have revealed that mutations within the S protein, particularly in the S1 subunit, can induce structural alterations that affect its stability and conformational dynamics, particularly the protein’s ability to switch between the open and closed states that can influence the accessibility of the RBD critical for viral attachment to host cells. Moreover, long-range interactions between the dynamic S1 subunit (including domains like the N-terminal domain and RBD) and the more rigid S2 subunit (involved in membrane fusion) play crucial roles in determining the overall architecture and functional states of the S protein trimer [16,17,18].

The emergence and evolution of SARS-CoV-2 variants like BQ.1.1 and XBB.1 have raised significant interest due to their distinct characteristics, including superior growth advantages and potential immune evasion capabilities [19,20]. The XBB.1.5 subvariant has evolved through recombination events within the BA.2 lineage, incorporating genetic material from BA.2.10.1 and BA.2.75 sublineages. XBB.1.5 is equally immune evasive as XBB.1 but may have growth advantage by virtue of the higher ACE2 binding as F486P in the XBB.1.5 subvariant can restore most of the favorable hydrophobic contacts [21]. Further investigations have substantiated that the enhanced growth and increased transmissibility observed in the XBB.1.5 lineage likely stem from its preserved resistance to neutralization and improved affinity for binding to ACE2 receptors [22]. By October 2023, XBB sublineages such as XBB.1.5 and XBB.1.16, both featuring the F486P substitution, had become prevalent globally (source: https://nextstrain.org/) (accessed on 5 February 2024) [23]. Compared to XBB.1.5, XBB.1.16 exhibits two substitutions: E180V in the NTD and T478R in the receptor-RBD [24]. These emerging variants demonstrated increased infectivity and transmissibility compared to earlier Omicron variants. Moreover, several residues in the RBD (R346, K356, K444, V445, G446, N450, L452, N460, F486, F490, R493 and S494) are mutated in at least five distinct new Omicron lineages. The lineage of XBB descendants, including EG.5 and EG.5.1, which carry an additional mutation F456L, has become one of the dominant lineages currently. EG.5 evolved from Omicron XBB.1.9 and bears only one additional substitution, F456L, compared to XBB.1.5. Its direct descendant, EG.5.1, features Q52H in the NTD and F456L in the RBD [25]. EG.5 and EG.5.1 were discovered to exhibit moderate resistance to antibody neutralization compared to XBB.1.5 and this resistance is particularly pronounced against class 1 monoclonal antibodies, primarily due to a single F456L mutation in the RBD [26]. Further studies specifically on the immune evasion of the EG.5.1 subvariant confirmed that the enhanced neutralization escape observed is primarily driven by the F456L mutation rather than the Q52H mutation [27]. XBB subvariants carrying both the L455F and F456L combination of flipped substitutions are often referred to as “FLip” variants and these variants were identified in over 20% of global XBB samples collected at the beginning of September 2023 [23]. The FLip variants include JG.3 (XBB.1.9.2.5.1.3.3), JF.1 (XBB.1.16.6.1), GK.3 (XBB.1.5.70.3) and JD.1.1, all of which emerged convergently. This convergence underscores that acquiring the L455F/F456L double mutation can provide a growth advantage to XBB within the human population [28].

**Figure 1:**
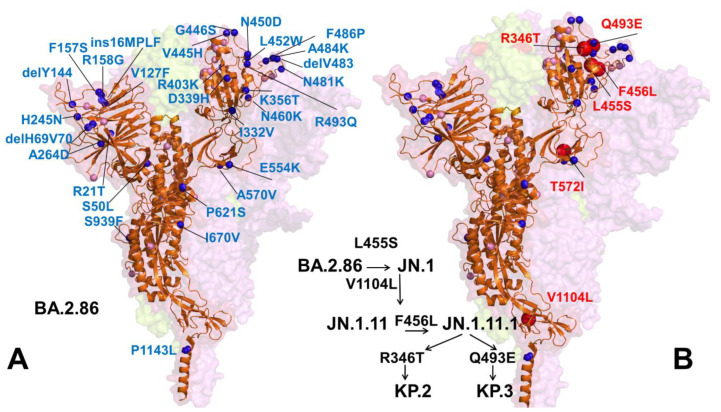
Structural overview of the SARS-CoV-2 S protein and S-RBD for Omicron BA.2.86 (**A**) and JN.1/KP.2/KP.3 variants (**B**). The S protein is shown on orange ribbons (single monomer) with the S protein trimer structure shown on the surface with reduced transparency. The BA.2.86 RBD mutations are projected onto crystallographic RBD conformation (orange ribbons) in the BA.2.86 RBD-ACE2 complex, pdb id 8QSQ. The positions of unique BA.2.86 S mutations relative to its ancestral BA.2 variant are shown in blue-colored spheres and fully annotated. BA.2 mutational positions are pink spheres. The unique JN.1, KP.2 and KP.3 S mutations in panel (**B**) are shown as red spheres, and BA.2.86 mutations are blue spheres and annotated. For presentation convenience and clarity, the ACE2 receptor is not shown.

The Omicron subvariant BA.2.86, originating from the BA.2 variant, shows substantial genetic divergence from earlier forms (Table 1, Figure 1) [29,30,31,32]. Biophysical investigations have confirmed that the BA.2.86 variant can resist neutralization by monoclonal antibodies targeting epitopes in the NTD and SD1, as well as classes 1, 2 and 3 epitopes within the RBD. Notably, BA.2.86 exhibits a greater potential to evade RBD-targeted antibodies compared to the immune evasion observed in XBB.1.5 and EG.5.1 variants [29]. The immune evasion capability of the BA.2.86 subvariant was evaluated using a panel of neutralizing antibodies that are effective against XBB.1.5, revealing that BA.2.86 can evade antibodies that target XBB.1.5 [30]. Recent studies investigating the structure and binding characteristics of the BA.2.86 spike protein with ACE2 and antibodies have shown that the mutations acquired by BA.2.86 do not result in significant changes in antibody evasion compared to XBB.1.5 [32]. However, the RBD of BA.2.86 displays a 2.2-fold increase in affinity for ACE2 compared to XBB.1.5, thereby providing BA.2.86 with a transmission advantage. The latest cryo-EM structure of ACE2 complexed with BA.2.86 trimeric S protein supported the notion that the enhanced binding affinity of BA.2.86 may be driven by the electrostatic complementarity between BA.2.86 RBD and ACE2 and also potentially enhanced by the flexibility of the RBDs, allowing better exposure of the ACE2 binding within the trimer structure [32]. Another study also suggested that BA.2.86 does not have greater immune escape relative to XBB.1.5 from neutralizing immunity elicited by either Omicron XBB-family subvariant infection but improves virus fitness through greater binding affinity to ACE2 [33].

JN.1 is a variant of BA.2.86 which emerged independently from Omicron BA.2 and harbors an additional L455S mutation responsible for enhanced immune escape [34]. BA.2.86/JN.1 also showed a long genetic distance from XBB.1.5 and XBB.1.16 (Table 1, Figure 1). The JN.1 variant is antigenically distinct from the XBB.1.5 variant. A comparative biochemical analysis using surface plasmon resonance (SPR) assays showed a notable reduction in ACE2-binding affinity for JN.1, indicating that its enhanced immune evasion capabilities come at the expense of reduced ACE2 binding [34]. Despite only a single additional mutation (L455S) compared to its predecessor BA.2.86, which results in increased resistance to humoral immunity, the JN.1 variant swiftly became predominant in Europe and outcompeted the previously dominant XBB lineage by early 2024 [34,35]. In vitro ACE2-binding assay showed that the dissociation constant value of the JN.1 RBD was significantly higher than that of the BA.2.86 RBD, indicating that L455S mutation leads to the decreased binding affinity while it displays robust immune resistance, particularly against antibodies induced by the XBB.1.5 vaccine [35]. JN.1 demonstrated increased evasion against RBD class 1 antibodies, such as S2K146 and Omi-18, and the class-3 antibody S309, corroborating findings from other related studies [28,34].

A series of variants with mutations at L455, F456 and R346 convergent hotspot sites emerged, including the “SLip” variant which has the JN.1 mutations (L455S) with the additional F456L mutation [36]. More recently, we have seen the emergence of the FLiRT variant, which harbors an additional R346T mutation in the backbone of SLip. Recent studies revealed that that F456L (SLip) and R346T (FLiRT) subvariants of JN.1 contribute to further escape of JN.1-derived variants from neutralizing antibodies [36,37]. Since January 2024, JN.1 has diversified into a number of sublineages, many of which share recurrent mutations R346T (JN.1.18), F456L (JN.1.16), T572I (JN.1.7) or combinations of these mutations (KP.2 variant). JN.1 subvariants, including KP.2 (JN.1.11.1.2) and KP.3 (JN.1.11.1.3), which convergently acquired S protein substitutions such as S:R346T, S:F456L, S:Q493E and V1104L, have emerged concurrently [38,39]. KP.2 and KP.3 are members of a “FLiRT” group of variants. Other FLiRT variants, including KP.1.1, have also been identified as circulating in the US, but have not yet become as widespread as KP.2 or KP.3 [38,39]. JN.1 subvariants with one or more of these recurrent mutations, such as KP.2 (R346T, F456L and V1104L), appear to have a growth advantage [38]. According to WHO Coronavirus Network (CoViNet) (https://data.who.int/dashboards/covid19/variants) (accessed on 10 May 2024) currently circulating COVID-19 variants of interest (VOIs) as of 5 June 2024 include the EG.5 lineage (XBB.1.9.2 + S:F456L that includes EG.5.1 (23F): EG.5 + S:Q52H, HK.3 (23H): EG.5 + S:Q52H, S:L455F and HV.1: EG.5 + S:Q52H, S:F157L, S:L452R); BA.2.86 (23I); JN.1 (24A). Currently circulating COVID-19 variants under monitoring (VUMs) as of May 2024 include JN.1.7 (JN.1 + S:T572I, S:E1150D); KP.2 (JN.1 + S:R346T, S:F456L, S:V1104L); KP.3 (JN.1 + S:F456L, S:Q493E, S:V1104L) and JN.1.18 (JN.1 + S:R346T). As of June 2024, current circulating variants in the US are dominated by KP.2.3 (9.4%), KP.2 (7.1%), KP.3.1 (6.2%) and KP.3.3 (4.8%). As of 11 June 2024, the SARS-CoV-2 Omicron variants KP.2 and KP.3 have high prevalence in the United States. According to CDC Nowcast (https://covid.cdc.gov/covid-data-tracker/#variant-summary) (accessed on 10 May 2024) the proportion of illnesses caused by KP.3 rapidly increased from 9.4% estimated during the week of 11 May to 25% estimated during the week of 11 June 2024.

Virological properties of KP.2 variants were examined, showing that that the infectivity of KP.2 is significantly lower than that of JN.1 and that KP.2 has an increased immune resistance ability compared to JN.1 [38]. Another JN.1 descendant termed the “FLuQE” variant (KP.3) continues to dominate “FLiRT” and show strong growth. KP.3 (JN.1.11.1.3) features mutations R346T, L455S, F456L, Q493E and V1104L [39]. The pseudovirus infectivity of KP.2 and KP.3 was significantly lower than that of JN.1. Furthermore, JN.1 subvariants such as LB.1 and KP.2.3, which convergently acquired S31 deletion in addition to the above substitutions, have emerged and spread as of June 2024 and contribute to immune evasion and the increased relative effective reproduction number [40]. Importantly, LB.1 and KP.2.3 exhibited higher pseudovirus infectivity and more robust immune resistance than KP.2, showing that S31del is important for the increased infectivity and enhanced immune evasion. Both KP.2 and KP.3 variants share the F456L mutation that is critical for enhancing antibody evasion by impairing binding to class 1 RBD antibodies, confirming that antibody evasion can represent a major selective advantage for viral spread [41]. According to recent findings from the Cao lab [41] and private communications of 4 June (https://twitter.com/yunlong_cao) (accessed on 10 June 2024) the KP.3 variant is starting to outcompete KP.2 due to Q493E mutation that enables the higher ACE2-binding affinity than KP.2. and enhanced immune evasion primarily against class 1 of RBD antibodies. Moreover, KP.3 (JN.1 + F456L + Q493E) is the most immune-evasive variant and is also the fastest-growing JN.1 sublineage. The additional F456L and Q493E mutation allows KP.3 to evade a substantial proportion of JN.1-effective antibodies, especially class 1 antibodies.

Deep mutational scanning (DMS) experiments and functional studies have indicated that evolutionary opportunities for Omicron variants may be amplified by interactions among variant mutations, where the effect of one mutation can be influenced by the presence of others. This results in non-additive effects of mutations on specific functions [42,43,44,45,46]. These experiments have demonstrated compensatory epistasis, wherein individual immune escape mutations may diminish ACE2 binding, but such effects can be counterbalanced by epistatic interactions with affinity-enhancing mutations like Q498R and N501Y [45,46]. Recent DMS experiments have further explored the impact of all mutational changes and single-codon deletions within the XBB.1.5 RBDs on ACE2-binding affinity and RBD-folding efficiency. These studies have unveiled the network of epistatic interactions among RBD residues, including notable interactions between R493Q and positions Y453, L455 and F456 [47]. It was also demonstrated through mutational surveys of Omicron variants up until BA.2.86 that Q493E is typically detrimental to ACE2 binding, often incurring up to a 10-fold loss in the binding affinity [47]. In recent correspondence, Starr and colleagues reported that when Q493E mutation is combined with L455S and F456L mutations of FliRT variants, the loss of binding affinity is reversed and instead showed the increased ACE2 binding for KP.3 (https://x.com/tylernstarr/status/1800315116929560965) (accessed on 5 July 2024). Moreover, Starr and colleagues reported on the results of human ACE2-binding assays on all combinations of L455S, F456L and Q493E mutations in the BA.2.86 background, revealing the reversal of the deleterious effect of Q493E in the background of L455S + F456L (https://x.com/tylernstarr/status/1800315116929560965) (accessed on 5 July 2024). The most recent reports from Cao’s lab showed that enhanced receptor ACE2-binding capabilities of the KP.3 variant may be enabled by a substantial synergistic effect of F456L and Q493E mutations. While F456L (K_D_ = 12 nM) and R346T + F456L (K_D_ = 11 nM) did not largely affect the hACE2-binding affinity of JN.1 (K_D_ = 13 nM), Q493E mutation of KP.3 substantially improved the receptor-binding affinity, showing K_D_ = 6.9 nM which indicates non-additive epistatic interactions between Q493E and other mutations of KP.3, particularly F456L in comparison with these pre-BA.2.86 variants [48]. This insightful study also shows that high affinity of KP.3 due to epistasis may enable the incorporation of A475V (this mutation is convergently observed in JD.1.1, HK.3.14, JN.4 and KP.2.3.1 subvariants) for further immune evasion, given the observed of only small reduction in ACE2-binding affinity for KP.3 + A475V (K_D_ is 22 nM) [48]. This study indicated that the high ACE2-binding affinity of the KP.3 variant may drive the rapid transmission and prevalence of this subvariant and its descendants, enhancing its potential to acquire additional immune-evasive mutations [47]. Several other recent studies confirmed that JN.1 displays lower affinity to ACE2 and higher immune evasion properties compared to BA.2.86, indicating that new emerging subvariants bearing Q493E mutation in the background of FLiRT variants could recover ACE2-binding affinity and continue to display the enhanced antibody evasion profile [49].

Computer simulations provided important atomistic and mechanistic advances toward understanding the dynamics and function of the SARS-CoV-2 S proteins [50,51,52,53,54,55]. A series of experimental and computational studies revealed that the SARS-CoV-2 S protein can function as an allosteric regulatory machinery that is controlled by stable allosteric hotspots to modulate specific regulatory and binding functions [56,57,58,59,60,61,62]. Our recent studies demonstrated that convergent Omicron mutations such as G446S, F486V, F486P, F486S and F490S can display epistatic couplings with the major stability and binding affinity hotspots which may allow for the observed broad antibody resistance [60] Analysis of conformational dynamics, binding and allosteric communications in the Omicron S protein complexes with the ACE2 host receptor characterized regions of epistatic couplings that are centered at the binding affinity hotspots N501Y and Q498R [61]. MD simulations and Markov state models systematically characterized conformational landscapes of XBB.1, XBB.1.5 Omicron variants and their complexes, showing that convergent mutation sites could control evolution allosteric pockets through modulation of conformational flexibility of functional RBD regions [62]. AlphaFold2-based structural modeling approaches were combined with all-atom MD simulations and mutational profiling of binding energetics and stability for prediction of dynamics and binding of the SARS-CoV-2 Omicron BA.2.86 spike variant with the ACE2 host receptor [63]. This study quantified the role of the BA.2 and BA.2.86 backgrounds in modulating binding free energy changes, revealing critical variant-specific contributions of the BA.2.86 mutational sites R403K, F486P and R493Q. AlphaFold predictions of multiple conformations with MD simulations identified important differences in the conformational landscapes and binding energetics of the XBB variants and revealed the mediating role of the Q493 hotspot in epistatic couplings between L455F and F456L convergent mutations [64]. The results of mutational scanning and binding analysis of the Omicron XBB spike variants with ACE2 and a panel of class 1 antibodies provided a quantitative rationale to the experimental evidence that epistatic interactions of physically proximal binding hotspots Y501, R498, Q493, L455F and F456L residues can determine strong ACE2 binding, while convergent mutational sites F456L and F486P are instrumental in mediating broad antibody resistance [65].

The success of AF2 in predicting single protein structures has been widely recognized. However, these methods face inherent limitations when it comes to predicting multiple functional conformations of allosteric proteins or accurately capturing the effects of single-point mutations, which can lead to significant structural changes. A conceptually important limitation of AF2 methods is that the models are trained on a dataset of stable proteins that maintain their folded structure at physiological temperatures. Consequently, it predicts the most likely folded structure without assessing whether the protein could become unstable upon certain mutational changes. Moreover, most proteins are only marginally stable, making them highly vulnerable to mutations that may cause them to unfold. When mutations lead to notable distortions in the native structure, the performance of AF2 predictions may markedly deteriorate [66,67] with only weak or no correlations between the output metrics of AF2 and changes in protein stability or functionality [68]. A more recent analysis of AF2 methods for predicting effects of point mutations using AF-Cluster and SPEACH_AF adaptations showed that functionally relevant structural changes in the mutational models can be obtained when mutations are introduced in the entire MSA as compared to only the input sequence [69]. This study suggested that AF2 can accurately predict changes due to mutations that do not lead to major distortion in the native structure but are mostly associated with moderate changes in the free energy of unfolding. On the other hand, a critical limitation of AF2 methods is that structural or energetic consequences mutations are more difficult to capture when mutations lead to significant folding defects as AF2 has learned about protein energy landscapes from information encoded in the MSAs and the folded stable structures that it was trained on. [69]. The latest studies demonstrated that AF2 may be able to predict single-mutation effects with moderate structural changes as the localized structural deformation between proteins differing by only 1–3 mutations was correlated across 3901 experimental and AF-predicted structures [70]. Moreover, AF2-predicted structures can encode fine details about the energy landscape including information on stability and destabilizing effects of single mutations that do not disrupt protein structure [71]. As the number of possible mutations far exceeds the data points used to train AF2 methods, it remains challenging to accurately capture the full range of mutation effects. This challenge is further compounded by the fact that critical changes in stability often stem from subtle structural alterations whereas many important disease-associated mutations are allosteric and could induce large structural transformations and ensemble redistribution.

The lack of molecular details on structure, dynamics and binding energetics of the FLiRT and FLuQE variants including primarily JN.1, KP.2 and KP.3 RBD binding with the ACE2 receptor and antibodies provides a considerable challenge that needs to be addressed to rationalize the experimental data and establish the atomistic basis for the proposed molecular mechanisms. We employed an integrative computational approach in which structure and conformational ensembles of the of the JN.1, KP.2 and KP.3 RBD-ACE2 complexes were first predicted using AlphaFold2 (AF2) methods [72,73] using a shallow multiple sequence alignment (MSA) approach [74,75,76]. Structural studies of Omicron mutations and their effect on RBD conformation suggested that these mutations, both individually and in combination, typically induce relatively moderate structural changes and preserve the folded RBD conformation, where mutational-induced effects are associated with modulation of mobility in the flexible RBD regions. This firm experimental foundation provided a strong basis for employment of AF2 predictions and binding free energy analysis where it is assumed that the mutations of studied Omicron variants do not lead to major distortion in the native structure but are mostly associated with changes in the free energy of unfolding.

The molecular mechanics/generalized Born surface area (MM-GBSA) approach is then employed for binding affinity computations of the Omicron RBD-ACE2 complexes. We combined mutational profiling of the RBD residues with the MM-GBSA approach for binding affinity computations of the RBD-ACE2 complexes across FLiRT subvariants BA.2.86, JN.1 (L455S), BA.2.86 + F456L, BA.2.86 + Q493E, BA.2.86 + F456L/Q493E, BA.2.86 + L455S/F456L (KP.1), BA.2.86 + L455S/Q493E, BA.2.86 + R346T/L455S/F456l (KP.2) and BA.2.86 + L455S/F456L/Q493E (KP.3). Using mutational profiling of the S residues we identify binding energy hotspots and quantify epistatic couplings between convergent mutational hotspots. We examine a hypothesis that the emerging new variants may induce epistasis patterns where structural stability of the RBD can promote evolvability in vulnerable regions by tolerating combinations of convergent mutations at L455, F456, Q493 positions that confer beneficial phenotypes. Consistent with the latest functional studies of FLiRT and FLuQE variants, we found that that the Q493E mutation may be epistatically coupled with the F456L mutation, resulting in the reversal of the detrimental effect of Q493E seen in other backgrounds. The results showed that, combining the AF2 predictions, conformational ensembles of RBD-ACE2 complexes with MM-GBSA computations of the RBD-ACE2 binding can produce robust quantitative analysis of binding mechanisms. In addition, we also conducted mutational scanning of the S complexes with several important class 1 monoclonal antibodies to quantify the effect of convergent Omicron mutations on immune escape and the mechanism underlying the balance between ACE2 binding and immune evasion of BA.2.86, JN.1, KP.2 and KP.3 variants. The panel of studied antibodies included RBD-targeting antibodies such as S2K146 [77], Omi-3 [78] Omi-18 [78], Omi-42 [78] as well as antibodies BD55-5514 (SA55) and BD55-5840 (SA58) [79,80] that bind to a different RBD epitope and are experimentally known to tolerate escape mutations in BA.2.8, JN.1, KP.2 and KP.3 variants. Structural analyses of BD-508, BD-236, BD-629, BD-604 and BD-515 that are class 1 antibodies and displayed a similar RBD-binding pose showed diverse interactions between antibodies and RBD and pointed to high diversity that RBD antibodies may exhibit [81]. We show that a group of convergent mutational sites Y453, L455 and F456 represent prevalent escape hotspots against class 1 antibodies as all modifications in these positions, particularly L455S and F456L, lead to a dramatic loss of binding interactions with the antibodies. A particular class of antibodies, F2 and F3 antibodies, compete with ACE2, and their binding is affected by T376, K378, D405, R408 and G504, corresponding to class 1/4 which includes BD55-5514 (also known as SA55) [82]. Here, we demonstrate that SA55 and SA58 antibodies avoid targeting convergent mutational sites and therefore can overcome immune evasion. The results suggest a mechanism in which convergent Omicron mutations can promote high transmissibility and antigenicity of the virus by controlling the interplay between the RBD stability and conformational adaptability, allowing for optimal fitness tradeoffs between binding to the host receptor and robust immune evasion profile.

## 2. Materials and Methods

### 2.1. AI-Based Structural Modeling and Statistical Assessment of AF2 Models

We employed the AF2 framework [72,73] integrated into ColabFold [83] to conduct structural predictions of RBD-ACE2 complexes for variants JN.1, KP.2 and KP.3. Various parameters, including MSA depths, were adjusted during the process. The default MSAs were randomly subsampled to create shallow MSAs containing as few as five sequences. Two AF2 parameters, max_seqs and extra_seqs, were set using the max_msa field format: max_seqs determines the number of sequences used in the row/column attention track, while extra_seqs dictates additional sequences processed by the main evoformer stack. Lower values promote more diverse predictions but can increase the occurrence of misfolded models. Prior studies have suggested that modifying MSA depth can aid in conformational sampling [74,75,76]. In this study, the MSA depth was adjusted by configuring AF2’s max_extra_msa and max_msa_clusters parameters to 32 and 16, respectively. Additionally, parameters such as num_seeds and num_recycles were manipulated to enhance output diversity. Specifically, we set max_msa to 16:32, num_seeds to 4, and num_recycles to 12. AF2 makes predictions using five models pretrained with different parameters and different weights. To augment data generation, we set num_recycles to 12, resulting in 14 structures produced for each model across recycling iterations (from recycle 0 to recycle 12), culminating in a refined final structure. Recycling involves iterative refinement, progressively enhancing the precision of each recycled structure. Thus, each of the AF2 models yielded 14 structures, totaling 70 structures across all models.

AF2 models were assessed based on predicted local distance difference test (pLDDT) scores, which estimate confidence in predictions on a per-residue basis using a scale from 0 to 100. These scores are derived from the fraction of predicted Cα distances that fall within expected intervals [72,73]. To validate the structural accuracy, the predicted models of RBD-ACE2 complexes for the BA.2.86 variant (pdb id 8QSQ) [32] were compared with recently released experimental structures. Structural alignment was conducted using TM-align [84], aiming to achieve an optimal superposition of the two structures. The TM-score was then calculated to quantify the overall accuracy of the model predictions. We also used the root mean square deviation (RMSD) superposition of backbone atoms (C, Cα, O and N) calculated using ProFit (http://www.bioinf.org.uk/software/profit/) (accessed on 20 May 2024).

### 2.2. Molecular Dynamics Simulations

The crystal and cryo-EM structures of the Omicron RBD-ACE2 complexes were sourced from the Protein Data Bank [85]. In the case of simulated structures, hydrogen atoms and missing residues were initially added and allocated using the WHATIF program’s web interface [86]. Missing regions underwent reconstruction and optimization using the template-based loop prediction approach of ArchPRED [87]. Side-chain rotamers were refined and optimized using the SCWRL4 tool [88]. The protonation states of all titratable residues in ACE2 and RBD proteins were predicted at pH 7.0 using Propka 3.1 [89,90]. Following this, atomic-level energy minimization of the protein structures was performed using composite physics and knowledge-based force fields, implemented through the 3Drefine method [91,92]. We considered glycans that were resolved in the structures. Then, 1 µs all-atom MD simulations were performed for the Omicron RBD-ACE2 complexes with the aid of the NAMD 2.13-multicore-CUDA package [93] and using CHARMM36 force field [94]. The initial setup of the 2 S-RBD complexes involved preparation using Visual Molecular Dynamics (VMD 1.9.3) [95] and the CHARMM-GUI web server [96,97], utilizing the Solutions Builder tool. Prior to solvation with TIP3P water molecules [98] in a periodic box extending 10 Å beyond any protein atom in the system, hydrogen atoms were added to the structures. All Na+ and Cl− ions were positioned at least 8 Å away from any protein atoms and from each other. MD simulations are typically conducted in an aqueous environment where the number of ions remains constant throughout the simulation, providing a minimally neutralized ion environment or salt pairs to match the overall salt concentration [99]. For each protein system, a two-stage minimization protocol was applied. Initially, a 100,000-step minimization was performed with constraints on all hydrogen-containing bonds and fixed protein atoms. Subsequently, a second stage involved a 50,000-step minimization with fixed protein backbone atoms, followed by an additional 10,000 steps without fixed atoms. Following minimization, the protein systems underwent equilibration steps with a gradual increase in system temperature, incrementing in 20 K steps from 10 K to 310 K. At each temperature step, a 1 ns equilibration was conducted, maintaining a restraint of 10 kcal mol^−1^ Å^−2^ on the protein Cα atoms. After removing restraints on protein atoms, an additional 10 ns of equilibration was performed. Long-range non-bonded van der Waals interactions were computed using a cutoff of 12 Å, with a switching function starting at 10 Å and reaching zero at 14 Å. Hydrogen atom constraints were managed using the SHAKE method. Simulations employed a leap-frog integrator with a 2 fs time step. Water molecule constraints were managed using the ShakeH algorithm in NAMD. Long-range electrostatic interactions were computed using the particle mesh Ewald method [100] with a 1.0 nm cutoff and fourth-order (cubic) interpolation. The simulations were conducted using an NPT ensemble with a Langevin thermostat and a Nosé–Hoover Langevin piston at 310 K and 1 atm. The Langevin thermostat utilized a damping coefficient (gamma) of 1/ps. The Nosé–Hoover Langevin piston method integrates elements of the Nosé–Hoover constant pressure technique [101] with piston fluctuation control implemented through Langevin dynamics [102,103]. A production simulation under NPT conditions was performed on equilibrated structures for 1 microsecond, maintaining a temperature of 310 K and a constant pressure of 1 atm.

### 2.3. Distance Fluctuation Stability and Communication Analysis

We utilized distance fluctuation analysis on simulation trajectories to derive residue-based rigidity/flexibility profiles. This involved calculating fluctuations in the mean distance between each pseudo-atom of a specific amino acid and those of other protein residues. These fluctuations were then converted into distance fluctuation stability indexes, which quantify the energy required for residue deformation during simulations [104,105,106]. The distance fluctuation stability index for a given residue was determined by averaging distances to all other residues across the simulation trajectory, calculated as follows:

(1) $$k_{i}=\frac{3k_{B}T}{\left\langle (d_{i}-\left\langle d_{i}\right\rangle {)}^{2}\right\rangle }$$

(2) $$d_{i}={\left\langle d_{ij}\right\rangle }_{j*}$$

$d_{ij}$ is the instantaneous distance between residue i and residue j, $\;\;\;k_{B}$ i is the Boltzmann constant, T = 300 K. ⟨ ⟩ denotes an average taken over the MD simulation trajectory and $d_{i}={\left\langle d_{ij}\right\rangle }_{j*}$ is the average distance from residue i to all other atoms j in the protein. The reciprocal of these fluctuations provides an effective force constant that characterizes the ease of displacing an atom relative to the protein structure.

### 2.4. Binding Free Energy Computations: Mutational Scanning and Sensitivity Analysis

We analyzed the binding epitope residues of the SARS-CoV-2 S RBD-ACE2 complexes using mutational scanning. Each residue within the binding epitope underwent systematic mutation to explore all possible substitutions, and we calculated corresponding changes in protein stability and binding free energy. To conduct this analysis, we utilized the BeAtMuSiC approach [107,108,109] which evaluates how mutations impact the strength of interactions at the interface and the overall stability of the complex. The binding free energy of the protein–protein complex is determined by comparing the folding free energy of the complex with the folding free energies of the individual protein-binding partners.

(3) $$\Delta G_{bind}=G^{com}-G^{A}-G^{B}$$

The change in the binding energy due to a mutation was then calculated as the following:

(4) $$\Delta \Delta G_{bind}=\Delta G_{bind}^{mut}-\Delta G_{bind}^{wt}$$

We leveraged rapid calculations based on statistical potentials to compute the ensemble-averaged binding free energy changes using equilibrium samples from simulation trajectories. The binding free energy changes were obtained by averaging the results over 1000 and 10,000 equilibrium samples for each of the studied systems.

### 2.5. Binding Free Energy Computations

We calculated the ensemble-averaged changes in binding free energy using 1000 equilibrium samples obtained from simulation trajectories for each system under study. Initially, the binding free energies of the Omicron RBD-ACE2 complexes were assessed using the MM-GBSA approach [110,111]. Additionally, we conducted an energy decomposition analysis to evaluate the contribution of each amino acid during the binding of RBD to ACE2 [112,113]. The binding free energy for the RBD–ACE2 complex was obtained using:

(5) $$\Delta G_{\mathrm{bind}}=G_{\mathrm{RBD}–\mathrm{ACE}2}-G_{\mathrm{RBD}}-G_{\mathrm{ACE}2}$$

(6) $$\Delta G_{bind,MMGBSA}=\Delta E_{MM}+\Delta G_{sol}-T\Delta S$$

where Δ*E_MM_* is total gas phase energy (sum of Δ*E_internal_*, Δ*E_electrostatic_* and Δ*Evdw*); Δ*Gsol* is sum of polar (Δ*G_GB_*) and non-polar (Δ*G_SA_*) contributions to solvation. Here, G_RBD–ACE2_ represents the average over the snapshots of a single trajectory of the MD RBD–ACE2 complex, G_RBD_ and G_ACE2_ correspond to the free energy of RBD and ACE2 protein, respectively.

The polar and non-polar contributions to the solvation free energy are calculated using a generalized Born solvent model and consideration of the solvent accessible surface area [114]. MM-GBSA is employed to predict the binding free energy and decompose the free energy contributions to the binding free energy of a protein–protein complex on a per-residue basis. The binding free energy with MM-GBSA was computed by averaging the results of computations over 1000 samples from the equilibrium ensembles. First, the computational protocol must be selected as “single-trajectory” (one trajectory of the complex) or “separate-trajectory” (three separate trajectories of the complex, receptor and ligand). To save on computational cost and to reduce the noise in the calculations, it is common that each term is evaluated on frames from the trajectory of the bound complex. In this study, we choose the “single-trajectory” protocol because it is less noisy due to the cancellation of intermolecular energy contributions. This protocol applies to cases where significant structural changes upon binding are not expected. Hence, the reorganization energy needed to change the conformational state of the unbound protein and ligand are also not considered. Entropy calculations typically dominate the computational cost of the MM-GBSA estimates. Therefore, it may be calculated only for a subset of the snapshots, or this term can be omitted [115,116]. However, for the absolute affinities, the entropy term is needed, owing to the loss of translational and rotational freedom when the ligand binds. In this study, the entropy contribution was not included in the calculations of binding free energies of the RBD-ACE2 complexes because the entropic differences in estimates of relative binding affinities for the JN.1, KP.2 and KP.3 Omicron mutants are expected to be small owing to small mutational changes and preservation of the conformational dynamics [115,116]. MM-GBSA energies were evaluated with the MMPBSA.py script in the AmberTools21 package [117] and gmx_MMPBSA, a new tool to perform end-state free energy calculations from CHARMM and GROMACS trajectories [118]. gmx_MMPBSA provides the user with several options, including binding free energy calculations with different solvation models (PB, GB or 3D-RISM), stability calculations, entropy corrections and binding free energy decomposition [118]. A topology file is needed in this case to generate the topology files in Amber format with all the terms for CHARMM force field. We use a parallel implementation that speeds up the calculation by assigning an equal number of frames to each thread (process). gmx_MMPBSA and MMPBSA.py use the parallel MPI version only to perform the calculations, and the rest of the process, including conversion of Amber topologies, mutation and division of the trajectories, occurs in a single thread. The maximum efficiency is achieved as the number of frames processed is a multiple of the number of threads started. The calculations were run on the Keck College High Performance Computing (HPC) cluster at Chapman University using General-Purpose Supermicro Intel nodes with 256 Gb of RAM each and 72 Intel Xeon cores each.

## 3. Results

### 3.1. Evolutionary and Phylogenetic Analysis of Differences between XBB, BA.2.86 and FLiRT Lineages

**Figure 2:**
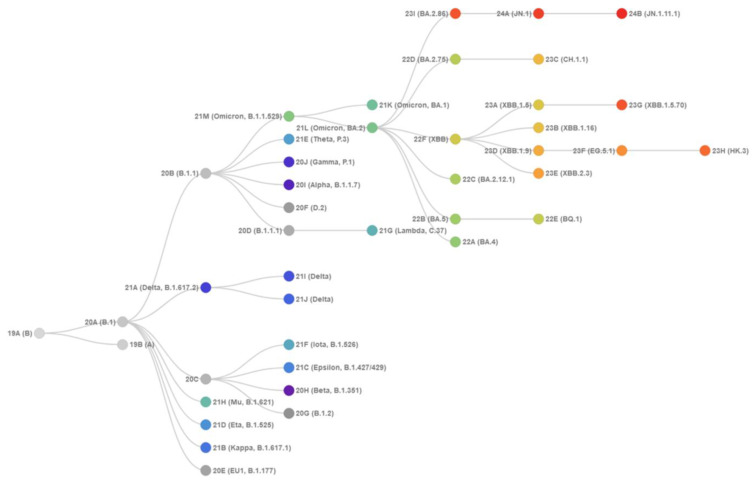
The evolutionary tree of current SARS-CoV-2 clades. XBB.1, XBB.1.5, BA.2.86, JN.1, jN.1.11.1 variants are shown on the current tree. The graph is generated using Nextstrain, an open-source project for real-time tracking of evolving pathogen populations (https://nextstrain.org/) (accessed on 5 February 2024) [39]. Clade 22F corresponds to XBB, 23A corresponds to XBB.1.5, 23I corresponds to BA.2.86, JN.1 corresponds to 24A and JN.1.11.1 corresponds to 24B clades. The evolutionary tree highlights divergence of BA.2.86 and JN.1 variants from XBB.1.5 lineage.

**Figure 3:**
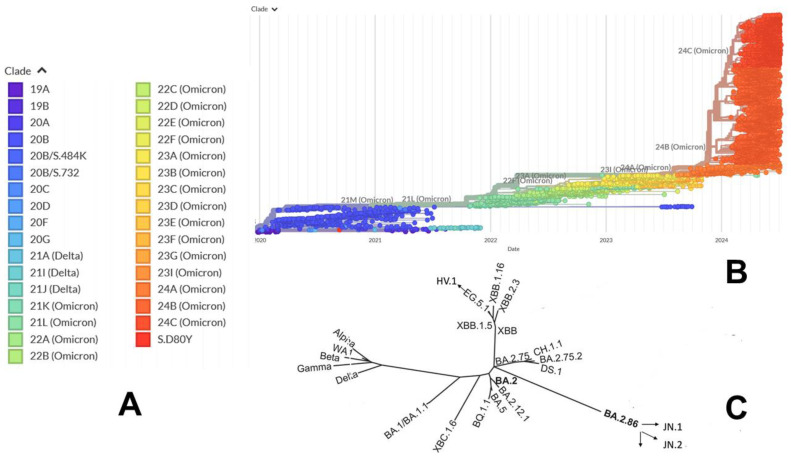
An overview of the phylogenetic analysis and divergence of Omicron variants. The graph is generated using Nextstrain, an open-source project for real-time tracking of evolving pathogen populations (https://nextstrain.org/) (accessed on 5 February 2024) (panels (**A**,**B**)). Phylogenetic analysis of SARS-CoV-2 clusters showing 7349 of 7349 genomes sampled between Dec 2019 and Jul 2024. BA.2.86 corresponds to Nextstrain clade 23I, JN.1 is clade 24A (BA.2.86 + S:L455S), JN.1.7 is clade 24A (J?N.1 + S:T572I, S:1150D), KP.2 is clade 24B (JN.1 + S:R346TK, D:F456L, S:V1104L) and KP.3 is clade 24C (JN.1 + S:F456L, S:Q493E, S:V110L). (**C**) The graph outlines diversification branches of Omicron mutations, highlighting evolutionary divergences between XBB.1.5 and BA.2.86 sublineages. Evolutionary trajectories of JN.1, JN.2 and JN.3 that originated from the BA.2.86 variant are schematically depicted.

The evolutionary divergence of the XBB and BA.2.86 lineages within the Omicron variants is depicted through phylogenetic analysis using their respective clade designations (Figure 2 and Figure 3) from Nextstrain, an open-source platform for real-time monitoring of pathogen evolution (https://nextstrain.org/) (accessed on 5 February 2024) [23]. Nextstrain offers interactive visualizations of the SARS-CoV-2 phylogenetic tree, facilitating the study of evolutionary relationships among different lineages and variants. This methodology assigns a variant as a clade when it reaches a global frequency of 20% at any given time, requiring each new clade to be at least two mutations distant from its parent major clade. The evolutionary analysis of Omicron variants highlights BA.2.86 as representing a significant evolutionary leap, providing the virus with a global growth advantage over the XBB.1-based lineages (Figure 2 and Figure 3).

BA.2.86 carries a unique constellation of mutations that have created a distinct branch in the SARS-CoV-2 spike phylogenetic tree (Figure 2 and Figure 3). BA.2.86 S exhibits a unique and distinct set of mutations that have led to the emergence of a new branch in the SARS-CoV-2 spike phylogenetic tree (Figure 2 and Figure 3). Compared to its ancestor BA.2, BA.2.86 carries 34 mutations, comprising 29 substitutions, 4 deletions and 1 insertion, which include notable changes in the RBD such as I332V, D339H, K356T, R403K, V445H, G446S, N450D, L452W, N460K, N481K, deletion of V483, A484K, F486P and R493Q (Figure 1). Several of these mutations, such as G446S, N460K, F486P and R493Q, have been previously identified in other variants. BA.2.86 is further classified into distinct sublineages BA.2.86.1, BA.2.86.2, BA.2.86.3, JQ.1, JN.1, JN.2, JN.3, JN.4, JN.5 and JN.6 [33,49,119]. The KP.2 (JN.1 + S:R346T, S:F456L, S:V1104L) group of lineages carries a signature mutation S:R346T. The KP.3 (JN.1 + S:F456L, S:Q493E, S:V1104L) group of lineages is characterized by a signature mutation S:Q493E (Figure 2 and Figure 3).

JN.1 emerges as a prominent descendant lineage of BA.2.86, classified under Nextstrain clade 24A (Figure 2 and Figure 3). By 2024, JN.1 had diversified into multiple sublineages, several of which share recurring mutations like R346T (JN.1.18), F456L (JN.1.16), T572I (JN.1.7) or combinations thereof, such as seen in the KP.2 variant. These mutations individually contribute to enhanced antibody evasion (R346T and F456L), ACE2-binding affinity (R346T) and stability and dynamic properties of the S protein (T572I) within the genetic context of earlier Omicron subvariants. Evolutionary diagrams illustrate distinct trajectories for the SARS-CoV-2 Omicron XBB and BA.2.86/JN.1 lineages (Figure 3). Current evolutionary divergences between XBB and BA.2.86/JN.1 lineages, as depicted in Nextstrain diagrams (Figure 3), suggest that Omicron lineages evolve through various mechanisms, including complex recombination, antigenic drift and convergent evolution. These processes have led to the independent acquisition of immune escape mutations such as R346T, L455F/S, F456L and T572I across multiple lineages.

### 3.2. AF2-Based Modeling and Prediction of the BA.2.86, JN.1, KP.2 and KP.3 RBD-ACE2 Complexes and Conformational Ensembles

In the current study we combined AI-based structural modeling approaches, MD simulations and the ensemble-based mutational profiling of the Omicron JN.1, KP.2 and KP.3 variants with the ACE2 receptor and a panel of monoclonal antibodies. AF2-based structural predictions of the top models for RBD-ACE2 complexes are combined with subsequent microsecond atomistic MD simulations to study the stability and dynamics of the AF2-predicted conformational states and characterize binding energetics of the RBD-ACE2 complexes. We first proceeded with AF2 structural predictions of the structures and conformational ensembles of the RBD-ACE2 complexes for the BA.2.86, JN.1, KP.2 and KP.3 variants using AF2 methodology [72,73] with varied MSA depth [74,75,76]. By using two AF2 parameters in the following format: *max_seqs:extra_seqs* we can manipulate the number of sequences subsampled from the MSA (*max_seqs* sets the number of sequences passed to the row/column attention track and *extra_seqs* the number of sequences additionally processed by the main evoformer stack). More diverse predictions are generally encouraged with the lower values and MSA depth adaptations may facilitate conformational sampling [74,75,76]. The predicted structural ensembles for the BA.2.86, JN.1, KP.2 and KP.3 variants revealed a considerable heterogeneity of the RBD in all variants, showing a progressive increase in the mobility in KP.2 and KP.3 (Supporting Information, Figure S1). Interestingly, for BA.2.86 and JN.1 variants, structural variations in the RBD loop 444–452 are rather moderate but become more diffuse in the KP.2 and KP.3 variants (Supporting Information, Figure S1). The RBD loop regions 444–452 and 475–487 harbor BA.2.86 mutations V445H, G446S, N450D, L452W, delV483, A484K and F486P. These regions are also in the immediate structural proximity of L455, F456 and Q493 positions that undergo mutational changes in JN.1, KP.2 and KP.3 variants.

**Figure 4:**
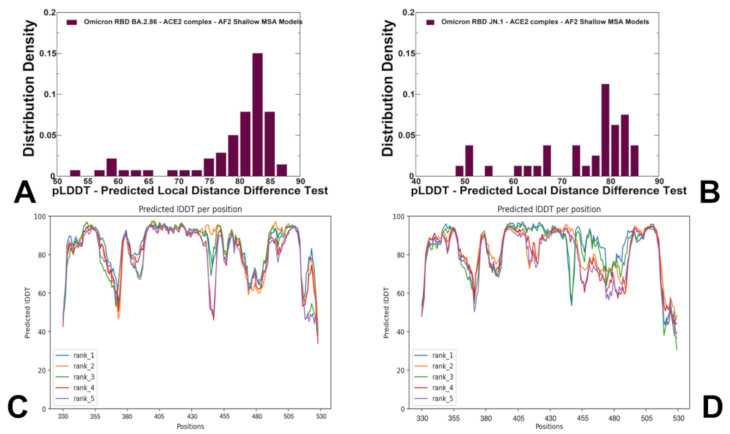
The distribution densities of pLDDT values and residue-based pLDDT profiles for the BA.2.86 and JN.1 RBD conformational ensembles obtained from AF2-MSA depth predictions. The density distribution of the pLDDT values for BA.2.86 (**A**) and JN.1 (**B**). The residue-based pLDDT profiles of the RBDN for BA.2.86 (**C**) and JN.1 (**D**) variants.

The statistical analysis and confidence evaluation of AF2 predictions are based on pLDDT scores, which are assigned to individual residues and indicate the model’s confidence in the accuracy of the predicted local structure. These scores measure the difference in distances between corresponding atoms in the predicted and reference structures. Ranging from 0 to 100, higher pLDDT scores reflect greater confidence in the local structure’s accuracy, with a score of 100 indicating high confidence, while lower scores suggest less certainty. The generated RBD conformations were evaluated using the pLDDT metric. The distribution densities of pLDDT values displayed pronounced peaks at pLDDT ~70–85 for studied variants BA.2.86 and JN.1 (Figure 4). The pLDDT assessments of the structural ensembles for the BA.2.86 variant (Figure 4A) showed that the dominant fraction of the ensemble (pLDDT ~80–85) conforms very closely with the experimental structure, while a minor fraction of the ensemble samples pLDDT values ~60–80 associated with the native RBD conformations but featuring the increased mobility of the RBD flexible loops (Figure 4A). Although other pLDDT distributions were generally similar, we noticed more shallow profiles for the JN.1 variant (Figure 4B). We also evaluated the residue-based pLDDT profiles that for most of the RBD residues ranged from 70 to 90, indicating fairly high confidence in the predictions (Figure 4C,D). The receptor-binding motif (RBM) is a crucial region (residues 438–506) of the RBD that directly interacts with the ACE2 receptor and plays a crucial role in determining affinity. The reduced pLDDT values (pLDDT ~55 to 70) were seen for the RBD loops (residues 381–394) and RBM tip region (residues 475–487) which is expected and consistent with the intrinsic flexibility of these RBD regions for all variants.

Notably, we observed small variation in the pLDDT profiles across the top five models for the BA.2.86 and JN.1 RBD-ACE2 complexes (Figure 4C,D). In some contrast, increased numbers of smaller peaks at various pLDDT values were seen for the KP.2 and KP.3 variants with a dominant distribution peak at pLDDT ~70–75 (Supporting Information, Figure S2). Moreover, the analysis of pLDDT profiles indicated a progressively increased dispersion between the AF2 models for KP.2 and KP.3 RBD conformations (Supporting Information, Figure S2). Overall, the AF2-predicted conformational ensembles for the RBD-ACE2 complexes revealed the increased heterogeneity in the KP.2 and especially KP.3 RBD variants which may potentially enable these variants to leverage a more mobile RBD structure to modulate and evade antibody neutralization.

**Figure 5:**
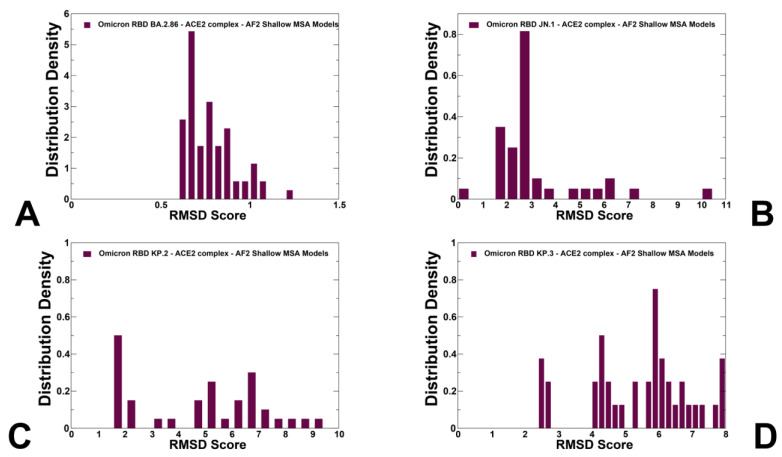
The density distribution of the RMSD score measuring structural similarity of the predicted RBD conformational ensembles with respect to the structure of the BA.2.86 RBD-ACE2 (pdb id 8QSQ) for the BA.2.86 (**A**) JN.1 (**B**), KP.2 (**C**) and KP.3 variants (**D**).

The distribution of structural similarity metric RMSD computed with respect to the cryo-EM structure of the BA.2.86 RBD-ACE2 complex echoed the pLDDT profile, showing strong peaks at low RMSD values ~1–2.5 Å for BA.2.86 (Figure 5A) and JN.1 variants (Figure 5B). Although the profiles showed low RMSD peaks for KP.2 and KP.3 variants, these distributions also possessed large RMSD values indicating the tendency of the AF2 predictions to produce overly heterogeneous conformations that could deviate significantly from the native structure of the BA.2.86 RBD (Figure 5C,D).

**Figure 6:**
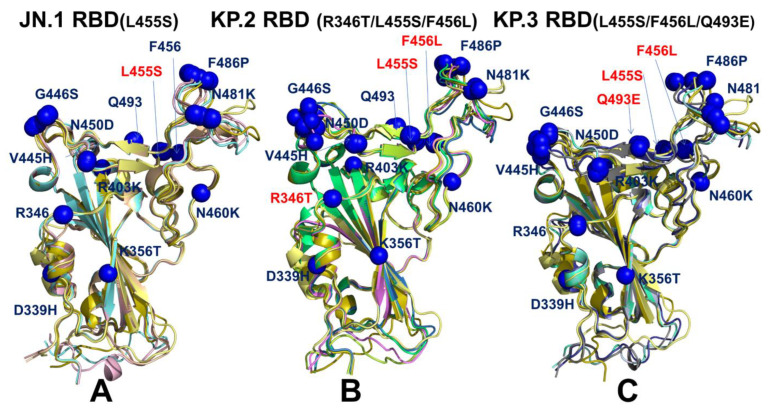
Structural alignment of the AF2-predicted RBD conformational ensembles in the complexes with ACE2 for JN.1 (**A**), KP.2 (**B**) and KP.3 variants (**C**). Structural alignment of the AF2-predicted conformations with high pLDDT values > 80.0 and the cryo-EM structure of the BA.2.86 RBD-ACE2 complex (pdb id 8QSQ). The experimental structure of the BA.2.86 RBD-ACE2 complex (pdb id 8QSQ) is shown as orange ribbons. The RBD conformations are shown as ribbons. The sites of unique BA.2.86 mutations D339H, K356T, R403K, V445H, G446S, N450D, L452W, N460K, N481K, A484K, F486P, R493Q are shown as blue spheres in panels (**A**–**C**). Mutation L455S in JN.1 is shown as red spheres in panel (**A**). The positions of KP.2 mutations R346T, L455S and F456L are shown as red spheres in panel (**B**). The positions of KP.3 mutations L455S, F456L and Q493E are shown as red spheres in panel (**C**).

To characterize functionally relevant conformations from the ensemble, the top predicted models for the RBD-ACE2 complexes were selected solely based on the confidence metric pLDDT by choosing conformations with pLDDT > 70 (Figure 6). It is assumed that that conformations with high pLDDT values could be considered as functionally relevant representatives of the RBD mobility. Structural alignment of the AF2 models with the recently released cryo-EM structure of the ancestor BA.2.86 RBD-ACE2 complex (pdb id 8QSQ) yielded the RMSD < 0.8–1.0 Å, mostly displaying moderate deviations in the RBD loops which are especially apparent in the RBM region bearing mutations F486P and N481K (Figure 6).

The structural alignment of the predicted conformational ensembles (Figure 6) revealed moderate heterogeneity of the flexible RBD loops while the RBD core remained largely rigid. Moreover, structural analysis of the AF2-predicted ensemble conformations with high pLDDT value indicated that the degree of mobility in the RBD loops, particularly the RBM, can remain fairly similar across all variants. However, we noticed that JN.1 which is the immediate descendant of the BA.2.86 variant displayed very small deviations from the BA.2.86 cryo-EM structure (Figure 6A), including RBD loop 444–452. This is also evident from mapping of the mutational positions in all predicted conformations, showing that V445H, G446S, Q493, L455S, F456 sites remain virtually in the same positions in the top predicted conformations (Figure 6A). The alignment of JN.1 RBD conformations revealed only moderate flexibility of the RBM which is reflected in minor lateral displacements of F486P and N481K positions (Figure 6A). We observed a generally similar pattern in the KP.2 RBD conformations where moderate variability was seen in V445H/G446S sites, but the predicted positions of N481K and F486P remain largely the same despite the overall mobility of the RBM region (Figure 6B). Interestingly, the RBD backbone conformations around L455S and F456L remain mainly rigid with only minor variations of the side chains. Hence, functional heterogeneity of the RBD conformations may remain largely similar, suggesting that the virus exploits convergent mutational sites to only moderately modulate ACE2-binding affinity, while the major role of these mutations is likely to enhance the immune evasion potential. A greater conformational heterogeneity of the functional RBD conformations was observed in the KP.3 RBD-ACE2 complex (Figure 6C). In this case, we found that the RBD loops 444–452 and 475–487 may display functionally relevant flexibility, where the RBB conformations and positions of F486P/N481K could slightly differ among top conformations (Figure 6C). Although the majority of mutational sites remain in similar positions in different conformations of the ensemble, some moderate flexibility associated with convergent mutations R346T, L455S, F456L and Q493E can be noted. These findings from AF2 predictions suggest that L455S, F456L and Q493E in JN.1, KP.2 and KP.3 variants can undergo functional displacements around their native positions.

### 3.3. MD Simulations and Structural Analysis of the Binding Interface Residues in the JN.1, KP.2 and KP.3 RBD-ACE2 Complexes

Although AF2 predicted ensembles of the RBD conformations can capture main dynamic signatures and flexibility of the RBD-ACE2 complexes, using AF2 predictions directly for characterization of the conformational landscape might be challenging since this is not a physically correct thermodynamic ensemble of states. MD simulations provide a more detailed and granular view of conformational flexibility and structural changes that are intimately connected with physical models of protein energetics. In this work, we employed top AF2-predicted models of the of the BA.2.86, JN.1, KP.2 and KP.3 RBD-ACE2 complexes as robust starting points for subsequent MD simulations and refinement to provide a more adequate and physically rigorous characterization of the conformational ensembles for the RBD-ACE2 complexes. These equilibrium ensembles were also later utilized for mutational scanning analysis and binding free energy calculations.

We analyzed MD simulation trajectories using a comparative analysis of the residue-based distance fluctuation stability indexes (Supporting Information, Figure S3). The adaptation of this approach for the analysis of rigid and flexible residues in the S proteins was detailed in our previous studies [60]. It is worth mentioning that, in this approach, the high values of distance fluctuation stability indexes point to structurally rigid residues (which is the opposite trend as compared to RMSD values) as they display small fluctuations in their distances to all other residues, while small values of this index would point to more flexible sites that display larger deviations of their inter-residue distances. Importantly, the variations of this parameter provide insights into global dynamic patterns and long-range fluctuations while RMSD profiles can characterize only local flexibility patterns.

The distributions showed that the local maxima for all RBD-ACE2 complexes are aligned with structurally stable and predominantly hydrophobic regions in the RBD core (residues 400–406, 418–421, 453–456) as well as key binding interface clusters (residues 495–505) that include binding hotspots R498 and Y501. The RBD positions associated with the high distance fluctuation stability indexes are F400, I402, Y421, Y453, L455, F456, Y473, A475 and Y489 (Supporting Information, Figure S3). The stability hotspots Y449, Y473 and Y489 are constrained by the requirements to maintain RBD stability and binding with the ACE2 host receptor. The profile minima are associated with the flexible RBD regions (residues 355–375, 381–394, 444–452, 455–471, 475–487). Despite a similar shape of the distributions for all Omicron RBD variants, the larger peaks were seen for the JN.1 distribution (Supporting Information, Figure S3). This implies that both the RBD core and ACE2-binding interface positions are more rigid in JN.1 and become somewhat more flexible in KP.2 and especially KP.3 variants. The profiles pointed to flexible RBD regions (residues 355–375, 381–394, 444–452, 455–471, 475–487) that are shared by all variants and are associated with low stability indexes. Of particular interest are the lowered distance fluctuation stability indexes in the RBD regions 440–460 and 470–487 for KP.2 and KP.3 (Supporting Information, Figure S3).

These observations are consistent with AF2 predictions of more flexible RBD conformations for the KP.2/KP.3 RBD variants. By mapping BA.2.86 mutational sites on the distributions, we noticed that mutated RBD positions are typically characterized by moderate stability indexes, indicating that Omicron mutations target conformationally adaptable regions in the RBD. Mutational positions N440K, V445P, G446S, N460K, F486P displayed low distance fluctuation stability indexes, which may indicate the presence of local mobility in these regions. Several important RBD-binding interface centers, such as L455 and F456, showed intermediate stability indexes, indicating that although these positions overall remain stable in all variants, these sites may undergo moderate fluctuations in their mutational forms containing L455S and F456L changes (Supporting Information, Figure S3). At the same time, R498, Y501 and H505 featured high stability indexes, reflecting a considerable rigidification of these residues due to strong interactions with ACE2.

**Figure 7:**
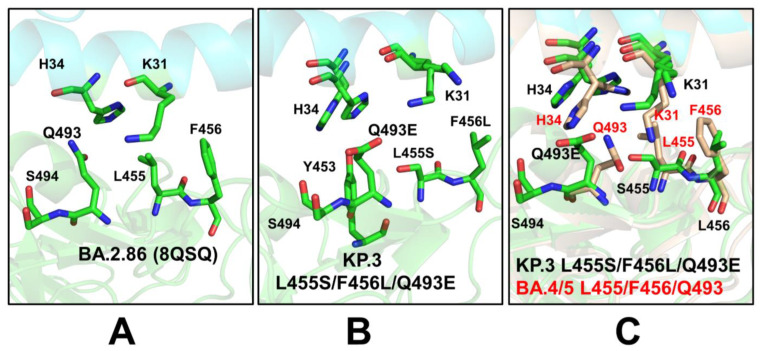
Structural predictions of the RBD-ACE2-binding interface. (**A**) A closeup of the AF2-predicted RBD-binding interface residues Q493, S494, L455, F456 and ACE2 K31 and H34 for the BA.2.86 RBD-ACE2 complex. The only ACE2 residues considered in this illustration are H34 and K31 (seen at the top of each panel). (**B**) A closeup of the AF2-predicted binding interface residues Q493E, S494, L455S, F456L and ACE2 K31, H34 for the KP.3 (L455S/F456L/Q493E) RBD-ACE2 complex. The AF2-predicted binding interface residues are atom-colored sticks. (**C**) A closeup of the binding interface residues Q493E, S494, L455S, F456L for KP.3 RBD-ACE2 complex (atom-colored sticks) overlayed on the experimental structure of the BA.4/5 RBD-ACE2 complex (pdb id 8H06). The binding interface residues S494, Q493, L455, F456, K31-ACE2 and H34-ACE2 of the BA.4/5 RBD-ACE2 complex are shown as brown sticks.

We first examined in some detail the predicted binding interfaces in the JN.1, KP.2 and KP.3 complexes. The binding interface in the cryo-EM structure of the BA.2.86 RBD-ACE2 complex (pdb id 8QSQ) showed that positions of H34 and K31 ACE2 residues as well as RBD residues Q493, L455 and F456 are very similar to those in the XBB.1.5 RBD-ACE2 complex [120] (Figure 7A, Supporting Information, Figure S4). In the XBB.1.5 RBD-ACE2 complex (pdb id 8WRL) the K31 side chain is placed between Q493 and hydrophobic L455 and F456 residues, showing that movements of H34 and K31 are fairly restricted (Supporting Information, Figure S4A). A similar interfacial pattern was predicted for the XBB.1.5 + L455F mutant where the favorable position of Q493 remained largely unchanged as compared to XBB.1.5 (Supporting Information, Figure S4B). Compared to XBB.1.5, the F456L mutation does not substantially affect the interactions on the RBD-ACE2 interface and yet Q493 and H34 undergo some rearrangements due to the increased conformational space which allows insertion of the H34 side chain between Q493 and S494 [120] (Supporting Information, Figure S4C). A similar interfacial pattern is further strengthened in the XBB.1.5 + FLip RBD-ACE2 complex (pdb id 8WRH) [120]. The flip of L455 and F456 provided synergistic reorganization of the interactions between H34 and Q493/S494 RBD residues (Supporting Information, Figure S4D).

This structural analysis also indicated that Q493, L455 and F456 positions may cooperate with the key binding affinity hotspot N501Y in modulating binding affinity with the ACE2 receptor. These results are broadly consistent with previous computational analysis and experimental data that quantified the effect of RBD mutations on the interactions with the ACE2 receptor, particularly showing that N501Y can result in one of the highest gains in ACE2 affinity conferred by RBD mutation [121]. A series of illuminating studies from Zheng’s laboratory combined atomic force microscopy (AFM)-based single-molecule force microscopy (SMFS) and MD simulations to show that the N501Y mutation introduced additional interactions that enhance ACE2 binding and plays a key role in the higher rate of transmission [122,123].

We examined the details of the predicted binding interface in the vicinity of L455 and F456 positions to illustrate the effect of mutations of L455 and F456 on the structural repacking induced by the L455F/F456L mutation and change in the binding contacts. Of special interest is the analysis of the binding interface in the KP.3 RBD-ACE2 complex, primarily to examine whether Q493E mutation can induce potential cooperative structural changes that may result in the observed epistatic improvement in the ACE2-binding affinity. Q493E involves the rarest of all nucleotide mutations, C->G, and occurs at a key residue. Intriguingly, Q493E marks another major change by reversing the basic trend in which the RBD interfacial region (438–506) is more basic, i.e., positively charged, in most of the Omicron variants. In fact, with V445H, N481K and A484K mutations in BA.2.86 this variant exemplified the largest increase in the positively charged RBD sites. Quite unexpectedly, Q493E in the KP.3 variant reverses this trend, making the RBM more acidic. We leveraged the conformational ensembles obtained from AF2 and MD simulations to map the most favorable interfacial interactions in the KP.2 RBD-ACE2 complex (Figure 7B). The second region of the RBD that includes important mutational positions R403, L455, Y453 and Q493 binds with the central segment of the α1 helix interface of the ACE2 interface residues (D30, K31, H34, E35 and D38). Structural analysis showed that both H34 and K31 ACE2 residues become more flexible in the complex as compared to XBB.1.5 and BA.2.86 complexes (Figure 7B). Moreover, we found that H34 and K31 can assume several preferential side-chain orientations that enable moderate structural rearrangements which allow more room for making stable hydrogen bond interactions with Q493Q (Figure 7B). In addition, it is observed that the H34 side-chain placements allow for stronger contacts with the Y453 residue.

The additional comparative analysis illustrated superposition of the predicted binding interface for the KP.3 RBD-ACE2 complex with the recently determined structures of the BA.4/5 RBD-ACE2 complex (pdb id 8H06) [124] (Figure 7C). Interestingly, the side chain of H34 adopts two alternative conformations in the BA.4/5 complex that are essentially identical to the preferential side-chain conformation of H34 in the KP.3 complex. H34 of hACE2 forms hydrogen bonding with Y453 of the BA.4/5 RBD and Q493 is hydrogen-bonded to K31 [124]. Similarly, the predicted K31 side-chain positions in the KP.3 complex are very similar to the experimentally determined K31 orientation in the BA.4/5 complex (Figure 7C). Hence, structural analysis of the ensemble-predicted binding interface for the KP.3 complex suggested the moderately increased flexibility of the ACE2 interfacial sites that are afforded by L455S/F456L mutations that reduced bulkiness of the hydrophobic packing and conferred room for the increased flexibility and improved interactions mediated by Q493E with H34 and K31 hotspots (Figure 7C). In addition, we found that Q493 lies in the structural proximity of RBD residues 447–457, 467–473, 484–484, 488–497. In particular, Q493E is close to residues 346 (R346T in FLiRT variants), 448–456 and the 483–484 region where BA.2.86 has ∆V483 and A484K.

These structural predictions were made prior to the emergence of the cryo-EM structure of JN.1 RBD in complex with human ACE2 (pdb id 8Y18) and accurately reproduced the experimentally observed conformational adjustments of the key interfacial residues. Consistent with this very latest study [125], our structural analysis showed that the R493Q reverting mutation in the BA.2.86 RBD can enhance RBD-ACE2 interactions, whereas the L455S mutation in the JN.1 RBD induces loss of van der Waals interactions and reduced the receptor-binding affinity. The computational structural analysis performed in this study and experimental studies of the JN.1 variant binding offers support to the notion that evolution of Omicron variants proceeds along a functional trajectory where immune escape is gradually enhanced, while ACE2-binding capacity can moderately fluctuate and remain sufficiently high to ensure strong receptor affinity [125].

### 3.4. Ensemble-Based Mutational Profiling of the Binding Interface on the JN.1, KP.2 and KP.3 RBD-ACE2 Complexes

Using conformational ensembles obtained from MD simulation studies of the RBD-ACE2 complexes for the BA.2.86, JN.1, KP.2 and KP.3 variants, we performed a systematic mutational scanning of the RBD residues in the RBD-ACE2 complexes. Mutational scanning of the key RBD positions in the cryo-EM structure of the BA.2.86 RBD-ACE2 complex (Supporting Information, Figure S5) was analyzed focusing on the RBD positions that correspond to sites of convergent evolution to determine changes seen in JN.1 (L455S), KP.2 (R346T, L455S, F456L) and KP.3 (L455S, F456L, Q93E). Most mutations produced large destabilization binding free energy values with the exception of Y453F that resulted in a negligible change (Supporting Information, Figure S5A). This is in full agreement with the experimental studies that demonstrated the role of Y453F as an adaptive mutation which increased virus interaction with the mink ACE2 receptor, without compromising its utilization of the human ACE2 receptor [126,127]. Mutational profiling at L455 showed that all modifications, particularly L455S, are appreciably deleterious for ACE2 binding (ΔΔG~1.0 kcal/mol) (Supporting Information, Figure S5B). Mutational changes at the F456 position of the BA.2.86 RBD-ACE2 structure are generally very unfavorable, leading to ΔΔG~1.5–2.0 kcal/mol. However, F456L mutational change alone in the genetic background of BA.2.86 is rather small (ΔΔG~0.3 kcal/mol) and within the error margin, indicating that even individually F456L cannot significantly decrease the ACE2-binding affinity (Supporting Information, Figure S5C). Of particular interest are mutations in the Q493 position showing relatively moderate destabilization for Q493R and Q493E (ΔΔG~0.6 kcal/mol) (Supporting Information, Figure S5D). These results agree with DMS studies that projected Q493E as a destabilizing mutation in the backgrounds of the XBB.1.5 variant. Our results suggested that F456L and Q493E may be only marginally deleterious and may reverse their effect on ACE2 binding.

**Figure 8:**
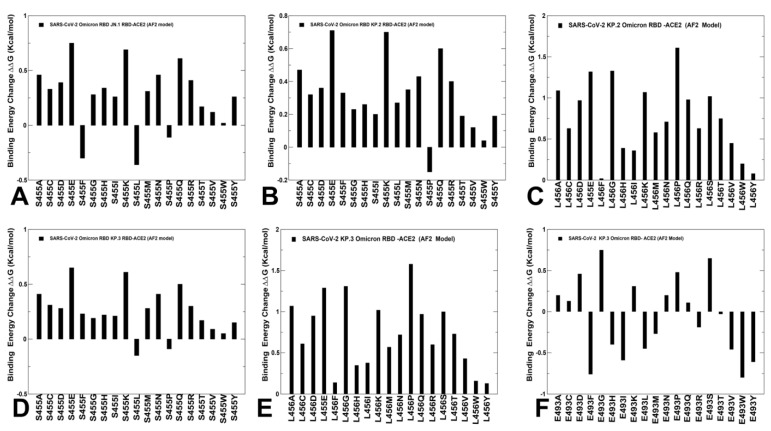
Ensemble-based mutational scanning of binding for the key RBD positions in backgrounds of JN.1, KP.2 and KP.3 variants. The conformational ensembles used in calculations are obtained from AF2 simulations and MD simulations of the RBD-ACE2 complexes for JN.1, KP.2 and KP.3 variants. The AF2-predicted structures were used as starting points to launch MD simulations. The profiles of computed binding free energy changes ΔΔG upon 19 single substitutions for S455 position in JN.1 (**A**), S455 in KP.2 (**B**), L456 in KP.2 (**C**), S455 in KP.3 (**D**), L456 in KP.3 (**E**) and E493 in KP.3 background (**F**). The respective binding free energy changes are computed using conformational ensembles obtained from AF2 predictions and MD simulations. The positive binding free energy values ΔΔG correspond to destabilizing changes and negative binding free energy changes are associated with stabilizing changes.

We then leveraged AF2 and MD conformational ensembles to perform ensemble-based mutational scanning of key RBD positions that are mutated in JN.1, KP.2 and KP.3 (Figure 8). Mutational analysis of S455 in the JN.1 RBD-ACE2 ensemble showed that mutations S455F, S455L and S455P are favorable and the reversed S455L change can potentially lead to some improvement in binding affinity (Figure 8A). This further confirmed that the L455S mutation can markedly decrease ACE2 binding which is consistent with the experimental data [34,35]. We then investigated more closely the effect of mutations in key sites of KP.2 (R346T/L455S/F456L) and KP.3 variants (S455, L456 and E493). The results showed that S455F, S455L and S455P remain favorable which implies that L455S substitution is deleterious for ACE2 binding in different genetic backgrounds of JN.1 and KP.3 (Figure 8B,D). Moreover, the L456 position in both KP.2 and KP.3 RBD-ACE2 complexes appears to be highly favorable as all substitutions lead to destabilizing, ΔΔG~0.5–2.0 kcal/mol (Figure 8C,E).

Mutational profiling of E493 in the KP3 RBD-ACE2 complex showed a more complex and diverse picture as a number of modifications can improve binding affinity (Figure 8F) which is consistent with the fact that E493 is generally a destabilizing change. However, in the KP.3 background, the reverse mutation E493Q appeared to be marginally destabilizing, i.e., E493 becomes slightly more favorable when combined together with KP.3 changes L455S and especially F456L (Figure 8F). Previous studies showed that salt bridge interactions in the BA.2 RBD formed by R493 with E37, E35 and D38 residues in ACE2 are partially lost in the BA.2.86 RBD-ACE2 complex [63]. In the BA.2.86 RBD-ACE2 complex Q493 forms stable interfacial contacts with D30, K31, N33, H34 and E35, including hydrogen-bonding interaction with the K31 side chain and the carboxyl group of E35 from ACE2 [63]. We showed that Q493E can partially rearrange the interactions with more flexible H34, E35 and K31 side chains. Notably, the salt bridge formed by R493 of BA.2 with E35 of ACE2 is replaced by Q493 in BA.2.75, BF.7, XBB.1 and BA.2.86 [63]. Although mutational profiling of E493 in the KP.3 ensemble showed that many changes could be stabilizing, the observed changes are small and are often stabilizing for small hydrophobic substituents (Figure 8F). Overall, the results suggested that Q493 is quite tolerant to modifications and can be mutated in different Omicron variants to balance ACE2 binding and immune evasion. These results are also consistent with the experiments showing that small hydrophobic amino acids for Q493 and aromatic amino acids for N501 can be highly enriched for ACE2 binding [121,122,123]. Our analysis also suggested that mutations R493/Q493/E493 in different Omicron variants including Q493E in the latest KP.3 can partially reconfigure the interaction network while preserving the convergent binding pattern of interactions which may enable evolutionary advantage through epistatic couplings between key hotspots at 456 and 493 positions on the RBD. While the exact binding free energy changes induced by mutations of RBD sites in the JN.1, KP.2 and KP.3 complexes with ACE2 are yet to be fully confirmed by experimental binding studies, the trend of the predicted changes strongly suggests relatively moderate variability in the ACE2 binding where the affinity of the virus for its receptor is already sufficient for high transmission [128]. These results provide support to the prevailing notion in which evolutionary changes of Omicron variants can proceed by targeting the increased immune evasion potential while maintaining the acceptable ACE2 binding for productive viral transmission.

### 3.5. MM-GBSA Analysis of the Binding Affinities for the XBB RBD-ACE2 Complexes

**Figure 9:**
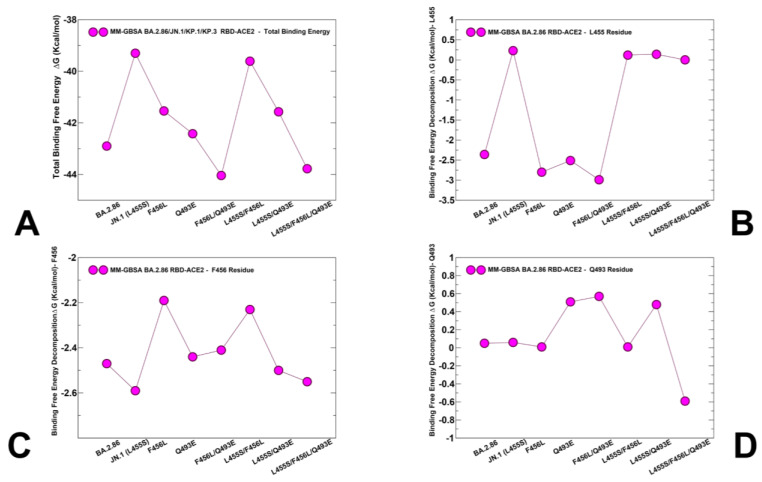
MM-GBSA binding free energies for BA.2.86, JN.1, BA.2.86 + F456L, BA.2.86 + Q493E, JN.1, BA.2.86 + F456L/Q493E, KP.2, BA.2.86 + L455S/Q493E and KP.3 RBD-ACE2 complexes. (**A**) The total binding free energies for the RBD-ACE2 complexes. (**B**) The MM-GBSA decomposition contribution of the binding energies for L455 position. (**C**) The MM-GBSA decomposition contribution of the binding energies for F456 position. (**D**) The MM-GBSA decomposition contribution of the binding energies for Q493E position.

According to the recent experimental studies using accurate SPR measurements the binding affinity of the JN.1 RBD-ACE2 complex is K_D_ = 13 nM, which is reduced as compared to that of the BA.2.86 variant (K_D_ = 1.7 nM), which is attributed to a deleterious L455S change in JN.1 [41]. At the same time, mutations F456L (K_D_ = 12 nM) and R346T + F456L (K_D_ = 11 nM) largely could not affect the ACE2-binding affinity of JN.1, indicating that the dampened ACE2 affinity of JN.1 due to L455S could not be fully compensated by F456L [41]. The central finding of this illuminating experimental study is that the Q493E mutation of KP.3 can markedly improve the ACE2-binding affinity, revealing K_D_ = 6.9 nM which is only marginally lower than the superior binding affinity of the BA.2.86 variant [41]. Using the conformational equilibrium ensembles obtained from AF2 predictions and MD simulations we computed the binding free energies for the BA.2.86, JN.1 (BA.2.86 + L455S), BA.2.86 + F456L, BA.2.86 + Q493E, BA.2.86 + L455S, BA.2.86 + F456L/Q493E, KP.2 (BA.2.86 + L455S/F456L/R346T) and KP.3 (BA.2.86 + L455S/F456L/Q493E) variants (Figure 9, Table 2).

The results of MM-GBSA computations showed a robust agreement with the experimental binding affinities (Figure 9). The total binding free energy changes showed a more favorable binding affinity for the BA.2.86 RBD-ACE2 with ΔG = −42.9 kcal/mol (Figure 9A). Consistent with the experiments, we found that L455S in JN.1 can lead to significant reduction in binding with ACE2 as compared to BA.2.86, showing ΔG = −39.3 kcal/mol (Figure 9A). As may be expected, the breakdown of the MM-GBSA binding energies (Table 2) showed the key role of the favorable van der Waals interactions (ΔG = −83.57 kcal/mol) in BA.2.86 that is markedly more favorable than that in JN.1 (ΔG = −79.20 kcal/mol). This MM-GBSA analysis confirmed that L455S in JN.1 can reduce the interaction energy and reduce the binding affinity. The energetic analysis of the BA.2.86 + F456L complex revealed that F456L alone has only a minor effect on the binding affinity, resulting in the overall ΔG = −41.54 kcal/mol (Figure 9A, Table 2) which is comparable to that of the BA.2.86 RBD-ACE2 complex. These results agree with recently reported unpublished observations by Starr and colleagues (https://x.com/tylernstarr/status/1800315116929560965) (accessed 25 June 2024).

According to MM-GBSA calculations, the binding affinity of the BA.2.86 + Q493E and especially BA.2.86 + Q493E/F456L complexes displayed a revealing and interesting trend in which the binding free energy is progressively improved to ΔG = −42.42 kcal/mol and ΔG = −44.04 kcal/mol (Figure 9, Table 2). Hence, our predictions indicate that coupling of Q493E and F456L mutations may fully restore binding affinity of the BA.2.86 variant. Interestingly, the MM-GBSA breakdown of the energetic components showed the reduced electrostatic contribution in variants bearing the Q493E mutation, but this reduction is compensated by the decreased polar solvation penalty, leading to the overall more favorable binding energies. The comprehensive MM-GBSA analysis is also in line with experiments [42] by demonstrating that binding affinity of the BA.2.86 L455S/F456L variant (ΔG = −39.61 kcal/mol) is similar to that of JN.1 (BA.2.86 + L455S) and therefore suggesting that F456L mutation alone cannot compensate for the loss of binding due to L455S.

The important finding of our analysis is that the KP.3 variant that bears L455S, F456L and Q493E displayed a considerably improved binding affinity (ΔG = −43.78 kcal/mol) as compared to the individual effects of F456L and Q493E alone (Figure 9A, Table 2). These results are consistent with the very latest experimental data showing that non-additive epistatic interactions between Q493E and other mutations of KP.3 can represent the energetic driver behind unexpectedly enhanced affinity of KP.3 [41].

To evaluate the contributions of L455, F456 and Q493 residues and corresponding mutations across all studied variants, we also reported the MM-GBSA residue-based breakdown (Figure 9B–D). We found that L455S can induce consistent and similar loss of binding interactions across all variants that share this mutation (Figure 9B), thus indicating that deleterious effect of L455S is not affected by the presence of F456L and Q493E mutations. Of particular interest is MM-GBSA decomposition analysis at the 456 position (Figure 9). We noticed that F456L can induce loss of binding interactions in BA.2.86 + F456L, while partly reversing the trend and restoring more favorable contribution in BA.2.86 + F456L/Q493E and KP.3 (BA.2.86 + L455S/F456L/Q493E) variants (Figure 9C). An overall similar trend is seen in the MM-GBSA decomposition of the Q493 position (Figure 9D) where small improvements in the contribution of the 493 position could be observed in the KP.3 variant. However, the differences between contributions of Q493/E493 in different variants are relatively minor. We also evaluated contributions of individual ACE2 positions H34 and K31 that are involved in interactions with L455, F456 and Q493 sites (Supporting Information, Figure S6). It can be seen that that BA.2.86 variants with F456L/Q493E and L455S/F456L/Q493E additions lead to a stronger contribution of the K31 position on ACE2 due to a favorable combination of van der Waals and electrostatic interactions (Figure 9). In BA.2.86, Q493 is hydrogen-bonded with K31 while, in F456L/Q493E and L455S/F456L/Q493E variants, E493 and K31 form strong ionic electrostatic interactions and there are more van der Waals contacts between Q493E and K31 of ACE2 (Supporting Information, Figure S6A). We also found that the contribution of H34 becomes more favorable in variants sharing F456L (Supporting Information, Figure S6B). Moreover, multiple side-chain conformations of H34 are involved in multiple contacts and form favorable interactions with Y453.

Together, structural and energetic analysis of BA.2.86 combinations including JN.1, KP.2 and KP.3 variants suggested that F456L and Q493E may act cooperatively in KP.3 to induce epistatic couplings and restore the binding affinity to the level of BA.2.86. Although precise molecular mechanisms underlying the observed epistatic effects of Q493E and F456L are likely to be complex, our results suggested that the increased flexibility of the RBD in the KP.3 complex may allow for more variability of the reciprocal ACE2 sites H34 and K31 that appeared to adopt multiple side-chain conformations in the structural ensembles. We found the side chain of H34 adopts two alternative conformations that are also seen in the BA.4/5 complex and forms hydrogen bonding with Y453 [124]. Structural examination of the predicted ensembles and MM-GBSA analysis of the RBD-ACE2 complexes for JN.1, KP.2 and KP.3 variants suggested that the flexibility of the binding interface can be properly exploited by Q493E, Y453 and F456L positions to enhance binding affinity. Overall, this analysis pointed to a central role of F456L as a potential regulator of epistatic couplings but also suggests a potential role of structurally proximal L452 and Y453 sites supporting the improved binding interfacial interactions in the KP.3 variant.

### 3.6. Mutational Profiling of Protein-Binding Interfaces with Distinct Classes of Antibodies

**Figure 10:**
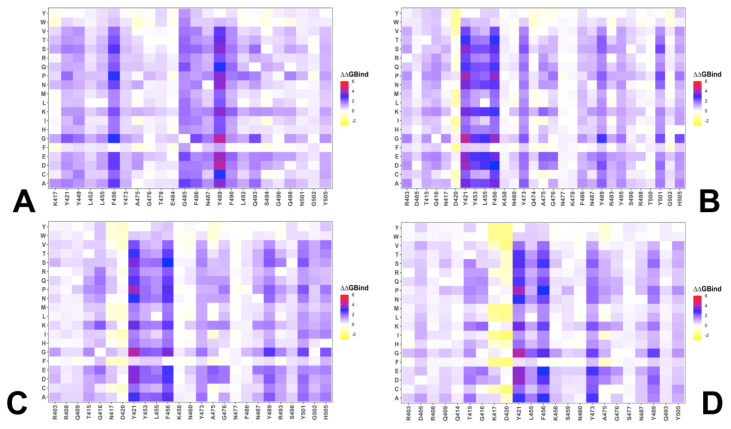
Ensemble-based dynamic mutational profiling of the RBD intermolecular interfaces in the Omicron RBD-ACE2 complexes. The mutational scanning heatmaps are shown for the interfacial RBD residues in the S complexes with S2K146 (**A**), Omi-3 (**B**), Omi-18 (**C**) and Omi-42 antibodies (**D**). The heatmaps show the computed binding free energy changes for 20 single mutations of the interfacial positions. The standard errors of the mean for binding free energy changes using 1000 randomly selected conformational samples (0.11–0.16 kcal/mol) were reduced to 0.08–0.12 kcal/mol when using 10,000 equally distributed samples from the MD trajectories.

We embarked on structure-based mutational analysis of the S protein binding with different classes of RBD-targeted antibodies, focusing specifically on the role of BA.2.86 mutations in mediating potential resistance to a broad class of antibodies and eliciting robust immune escape. We specifically examined a panel of monoclonal antibodies that were reported to retain activity against BA.2, XBB.1.5 and BA.2.86 variants but showed reduced neutralization against JN.1, KP.2 and KP.3 variants [41]. Structure-based mutational scanning of the S-protein-binding interfaces is carried out with a panel of the class 1 RBD-targeting antibodies S2K146 [77], Omi-3 [78] Omi-18 [78], Omi-42 [78] as well as antibodies BD55-5514 (SA55) and BD55-5840 (SA58) [79,80]. The results were compared with biochemical measurements from a panel of broadly neutralizing monoclonal antibodies [34,40,41]. To provide a systematic comparison, we constructed mutational heatmaps for the RBD interface residues of the S complexes with S2K146 (Figure 10A), Omi-3 (Figure 10B), Omi-18 (Figure 10C) and Omi-42 antibodies (Figure 10D). Strikingly, in all S complexes with class 1 antibodies mutational heatmaps clearly showed that a pair of adjacent residues, L455 and F456, corresponding to convergent evolutionary hotspots are also dominant escape hotpots of antibody neutralization. These results are consistent with the experimental data showing that antibody evasion drives the convergent evolution of L455F/S and F456L, while the epistatic shift caused by F456L can facilitate the subsequent convergence of L455 and Q493 changes to restore ACE2 binding [41]. Furthermore, mutational heatmaps indicated that other important positions involved in JN.1, KP.2 and KP.3 variants, particularly the Q493 site, can be considered as a secondary escape hotspot site mutations of Q493 across all class 1 antibodies (Figure 10). Interestingly, most of the RBD residues that directly bind S2K146 are also involved in binding to ACE2. Indeed, the mutational heatmap of the RBD binding against S2K146 identified that all substitutions in key interfacial positions can incur a consistent and considerable loss in binding affinity with S2K146 (Figure 10A). Among these sites are F456, F486, N487, Y489, F490 and Q493 as mutations in these sites caused the largest losses in the binding affinity with S2K146 (Figure 10A).

These findings are consistent with structural studies showing that the S2K146 footprint on the SARS-CoV-2 RBD mimics that of the ACE2 receptor, with 18 of 24 epitope residues shared with the ACE2-binding site, including L455, F486, Q493, Q498 and N501 [77]. The important finding of the mutational heatmap analysis is that positions Y421, Y453, L455 and F456 emerged as key escape hotspots in S binding with Omi-3, Omi-18 and Omi-42 class 1 antibodies (Figure 10B–D). L455 and F456 positions are located at the epitope of RBD class 1 antibodies and neutralization assays demonstrated that L455S mutation enables JN.1 to evade class 1 antibodies [34]. Mutational heatmap data showed that effectively all modifications in L455, including L455S, can cause considerable loss in antibody binding. In addition, the results also highlighted the importance of the Y453 position that is involved in favorable interactions with ACE2 and antibodies. A secondary group of escape hotspots for these class 1 antibodies included F486, N487, Y489 and Q493 positions (Figure 10B–D). Mutations in some of these sites such as F486P are implicated as the main immune escape hotspots for the BA.2.86 variant. Finally, due to mimicry of the ACE2 binding, other escape positions correspond to the ACE2-binding affinity hotspots Y501 and H505 (Figure 10). Importantly, the result suggested that major drivers of immune escape for this class 1 antibodies correspond to L455 and F456 sites that undergo mutations in the JN.1, KP.2 and KP.3 variants, enabling evolution through the enhanced immune escape.

**Figure 11:**
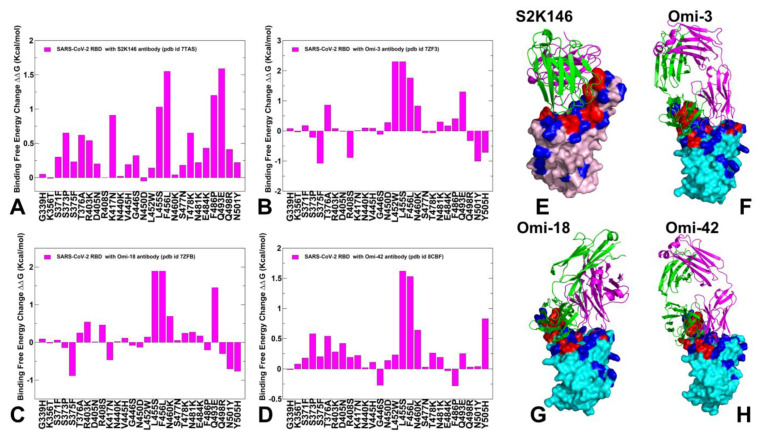
Structure-based mutational profiling of the S-RBD complexes with class 1 RBD antibodies. The mutational screening evaluates binding energy changes induced by BA.2.86/JN.1/KP.2/KP.3 mutations in the RBD–antibody complexes. Mutational profiling of the S-RBD complex with S2K146, pdb id 7TAS (**A**), S-RBD Omicron complex with Omi-3, pdb id 7ZF3 (**B**), S-RBD Omicron complex with Omi-18, pdb id 7ZF8 (**C**) and S-RBD in complex with Omi-42, pdb id 8CBF (**D**). The binding free energy changes are shown as magenta-colored filled bars. The positive binding free energy values ΔΔG correspond to destabilizing changes and negative binding free energy changes are associated with stabilizing changes. The 3D structures of the RBD–antibody complexes are shown for RBD-S2K146 (**E**), RBD-Omi-3 (**F**), RBD-Omi-18 (**G**) and RBD- BD-515 complexes (**H**). The RBD is shown in cyan-colored surface representation. The RBD-binding epitope residues are shown in red and BA.2.86 mutational positions are shown in blue. The antibodies are shown as ribbons (heavy chain in magenta and light chain in green-colored ribbons).

We then examined JN.1, KP.2 and KP.3 mutations using BA.286 as a genetic background to quantify binding free energy changes of S binding with the class 1 antibodies (Figure 11). The binding free energy changes associated with BA.2.86 mutations in the complex with S2K146 (Figure 11A) showed an appreciable loss of binding upon K417N, L455, F456L, F486P, Q493E and Q498R mutations. Interestingly, the largest destabilization changes were induced by L455S (ΔΔG = 1.03 kcal/mol), F456L (ΔΔG = 1.55 kcal/mol) and Q493E (ΔΔG = 1.59 kcal/mol). Hence, these key mutational changes present in JN.1, KP.2 and KP.3 variants may induce progressively enhanced immune escape from S2K146 which is consistent with experiments [34,40,41]. We found that L455S and F456L mutations induce very significant losses in antibody binding with Omi-3, Omi-18 and Omi-42 antibodies (Figure 11B–D). In particular, for binding with Omi-3, the L455S mutation caused ΔΔG = 2.3 kcal/mol and F456L incurred ΔΔG = 1.76 kcal/mol (Figure 11B), while for Omi-42 these losses were ΔΔG = 1.62 kcal/mol for L455S and ΔΔG = 1.53 kcal/mol for F456 (Figure 11D). These results are consistent with experiments showing the increased evasion against the JN.1 variant with Omi-3 and Omi-18 antibodies [34,40,41] as L455 and F456 sites emerge as dominant hotspots where mutations L455S and F456L caused the largest loss in affinity.

Common to both S2K146 and Omi-3 antibodies, we also observed a considerable loss of binding due to F486P mutation (Figure 11A,B). Specific for the Omi-3 antibody is a destabilizing role of the N460K mutation inducing loss of binding of ΔΔG = 0.83 kcal/mol (Figure 11B). Interestingly, Q493E emerges as the third most important escape mutation in binding with Omi-3 and Omi-18 antibodies (Figure 11B,C). Structural mapping of the binding epitope residues and sites of BA.2.86 mutations (Figure 11E–H) highlighted similar binding modes for these antibodies, targeting the second region of the RBD that includes R403, L455, Y453 and Q493 sites. In general, the results confirmed the experimental finding that KP.2 and KP.3 harboring the F456L mutation can significantly impair the neutralizing activity of RBD class 1 monoclonal antibodies such as BD-1854, BD57-1302 and Omi-42 [40].

**Figure 12:**
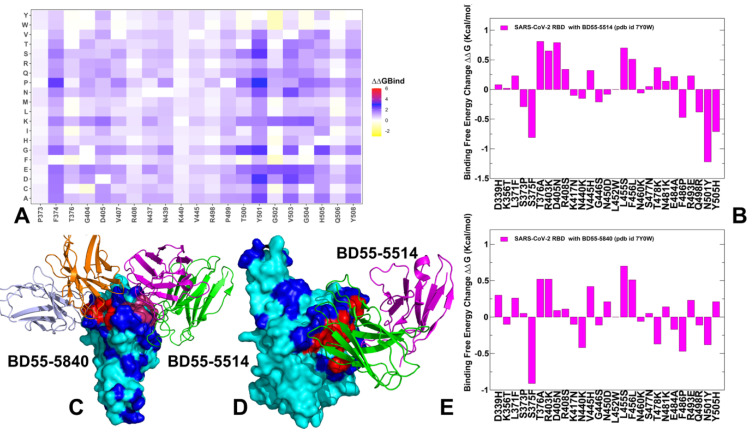
Structure-based mutational profiling of the S complexes with BD55-5840 (SA58; class 3) and BD55-5514 (SA55; class 1/4) antibodies. The mutational screening evaluates binding energy changes induced by BA.2.86/JN.1/KP.2/KP.3 mutations in the RBD–antibody complexes. (**A**) The mutational heatmap of the BD55-5514 interactions with the S protein shows the computed binding free energy changes for 20 single mutations of the interfacial positions. (**B**) Mutational profiling of the S complex with BD55-5514, pdb id 7Y0W. (**C**) The structure of the S complex with BD55-5840 and BD55-5514. (**D**) The structural view of the S protein bound to BD55-5514 in the same complex. The RBD is shown in cyan-colored surface representation. The RBD-binding epitope residues are shown in red and BA.2.86 mutational positions are shown in blue. The antibodies are shown as ribbons (heavy chain in magenta and light chain in green-colored ribbons). (**E**) Mutational profiling of the S complex with BD55-5840, pdb id 7Y0W. The binding free energy changes are shown as magenta-colored filled bars. The positive binding free energy values ΔΔG correspond to destabilizing changes and negative binding free energy changes are associated with stabilizing changes.

BD55-5514 belongs to class F2 and F3 antibodies that compete with ACE2, and their binding is affected by T376, K378, D405, R408 and G504 [79,80]. BD55-5514 displayed high potency against the Omicron subvariants and the recently developed non-competing antibody cocktail of BD55-5840 (also known as SA58; class 3) and BD55-5514 (also known as SA55; class 1/4), displayed high potency against the Omicron subvariants [125]. Recent studies by the Cao group showed that BD55-5514 (SA55) antibody can retain neutralizing efficacy against most of the known Omicron variants, including JN.1 [34]. SA55 antibody also showed significant neutralization activity against other variants sharing F456L mutation including HV.1 (L452R + F456L) and JD.1.1 (L455F/F456L + A475V) that typically greatly increase antibody evasion for other class 1 antibodies at the cost of ACE2 binding. The detailed mutational heatmap of the BD55-5514 interactions with the S protein showed a very different picture as compared to class 1 antibodies such as Omi-3, Omi-18 and Omi-42 (Figure 12A). The map showed that the S373P and S375F mutations could promote the interaction with BD55-5514. T376, D405 and R408 are involved in the interaction with BD55-5514 but they are all located at the periphery of the BD55-5514 epitope, and mutations in these positions have a moderate effect on binding affinity (Figure 12A). Importantly, the BD55-5514-binding interface does not involve escaping hotspot positions of BA.2.86, JN.1 and KP.2/KP.3 such as L455, F456 and Q493 (Figure 12A). A more detailed profiling of JN.1/KP.3 mutations against BD55-5514 antibody showed only small destabilization changes upon mutations T376A (ΔΔG = 0.81 kcal/mol), R403K (ΔΔG = 0.65 kcal/mol), D405N (ΔΔG = 0.79 kcal/mol), R408S (ΔΔG = 0.34 kcal/mol), L455S (ΔΔG = 0.7 kcal/mol) and F456L (ΔΔG = 0.51 kcal/mol) (Figure 12B). These changes reflect mostly a mild loss in the RBD stability and binding interactions, which is consistent with functional experiments showing that group F3 antibodies, such as BD55-5514, are not sensitive to the D405N and R408S mutations of BA.2, making this antibody effective against a broad spectrum of recent variants from BA.2.86 to KP.2 and KP.3 [129].

Structural mapping of the binding epitopes and BA.2.86 mutational sites for BD55-5514 and BD55-5840 antibodies (Figure 12C,D) illustrated how BD55-5514 can bind to the RBD together with BD55-5840 without interference. Mutational analysis of the BD55-5514 and BD55-5840 interactions confirmed that S373P and S375F mutations can improve the binding affinities of these antibodies with the S protein which is also consistent with the pseudo-virus data [74]. We also found that binding of BD55-5840 antibody is even less affected by mutations T376A, R403K and D405N as compared to BD55-5514 (Figure 12E). Importantly, both L455S and F456L mutations are only marginally deficient for binding of these antibodies with ΔΔG~0.5–0.7 kcal/mol (Figure 12A,E). As a result, these antibodies could potently bind to the entire spectrum of BA.2.86, JN.1, KP.2 and KP.3 mutations.

Our data provided a quantitative energetic analysis of the binding interactions for these antibodies in the complex where BD55-5840 and BD55-5514 act synergistically and bind to different sides of the RBD (Figure 12C). Interestingly, structural mapping of the BA.2.86 mutational sites onto the RBD complex with BD55-5514/BD55-5840 highlighted that binding modes of antibodies do not significantly overlap with the mutational positions (Figure 12C).

Together, structural and energetic analysis provides a rationale to the experimental results showing that the BD55-5840 (SA58) + BD55-5514 (SA55) cocktail exhibits remarkable neutralization breadth and potency [129] and particularly SA55 can retain neutralizing efficacy against all examined variants BA.2.86, JN.1, KP.2 and KP.3 [34]. A very recent study introduced CYFN1006-1 and CYFN1006-2 antibodies that demonstrated consistent neutralization of all tested SARS-CoV-2 variants comparable to or even superior to those of SA55 [130]. CYFN1006-2 exhibited high potency against all SARS-CoV-2 variants, with a slightly reduced efficacy against KP.2 [130]. These antibodies have binding epitopes that overlap with LY-CoV1404, REGN10987 and S309 that are situated on the outer surface of RBD and bind to a different RBD region compared to SA55. Hence, a cocktail with combinations of SA55 and CYFN1006-1 may be potentially beneficial against JN.1, KP.2, KP.3 and evolving mutants of SARS-CoV-2 [130].

## 4. Discussion

We examined quantitative aspects of structure, dynamics and energetics of the BA.2.86, JN.1 and KP.2/KP.3 RBD-ACE2 complexes by using an array of synergistic computational tools, including AF2 structural predictions of mutational effects and MD simulations. It is crucial to highlight that AF2 predictions are based on deep learning from experimentally determined structures. Although this approach can yield highly accurate static structures and top-ranked conformations, AF2 predictions are not sampled according to the Boltzmann thermodynamic probability distribution. As a result, the ensemble of structures generated by AF2 do not inherently represent the thermodynamic equilibrium ensemble of conformations, which would be weighted by their thermodynamic probabilities. While subsampling of the MSA and other AF2 enhancements can generate ensembles of protein conformations, some of the functionally relevant conformational states for proteins with complex and rugged landscapes can be overlooked. Using the predicted structural ensembles as starting structures for MD simulations can partially address these challenges, but the AF2-generated bias towards thermodynamically dominant states can hamper the robust predictions of the conformational landscapes. The experimentally observed conservation of the RBD fold and RBD-ACE2 binding interfaces across known SARS-CoV-2 S variants provided a firm experimental basis for robust AF2 modeling and prediction of mutational effects. Structural studies on Omicron mutations and their effects on RBD conformation have shown that these mutations typically result in only moderate structural changes, preserving the folded RBD conformation, where mutational effects are largely related to changes in the mobility of the flexible RBD regions. This experimental foundation strongly supports the application of AF2 predictions and binding free energy analysis, based on the assumption that Omicron variant mutations do not significantly distort the native structure. Hence, combining AF2 predictions with MD simulations offers a synergistic approach in which AF2 provides multiple accurate structures of the BA.2.86 RBD and ACE2 complexes, while MD simulations leverage these predicted conformations to characterize dynamics and energetics of binding with ACE2 receptor. Complementary to AF2-guided structural predictions, MD simulations revealed the details of the conformational landscape and the dynamic effect of BA.2.86, JN.1, KP.2 and KP.3 RBD mutations on binding to ACE2.

Recent studies of emerging Omicron variants, particularly evolving the BA.2.86 sublineage, suggested that the evolutionary paths for significant improvements in the binding affinity of the Omicron RBD variants with ACE2 are relatively narrow and may involve convergent mutational hotspots to primarily optimize immune escape while retaining sufficient ACE2 affinity. These mechanisms based on convergent adaptation may determine the scope of “evolutionary opportunities” for the virus to adapt new mutations that improve immune resistance without compromising ACE2 binding affinity and stability. The results of this study provided molecular rationale and support to the experimental evidence that functionally balanced substitutions that optimize tradeoffs between immune evasion, high ACE2 affinity and sufficient conformational adaptability might be a common strategy in virus evolution and serve as a primary driving force behind the emergence of new Omicron subvariants, including but not limited to BA.2.86, JN.1, KP.2 and KP.3 variants. The results of our investigation suggested the existence of epistatic interactions between convergent mutational sites at L455, F456, Q493 positions that enable protection and restoration of ACE2-binding affinity while conferring beneficial immune escape. Consistent with the latest functional studies, our results also showed that Q493E and F456L can act cooperatively through epistatic couplings to reverse the detrimental effect of individual Q493E mutation seen in other genetic backgrounds. Our results suggested that epistatic interactions between these sites may arise due to the increased side-chain flexibility of the interacting F456L, Q493E on RBD with H34 and K31 on ACE2 within a rather confined RBD-ACE2 interface. The progressively increased flexibility of the RBD-ACE2 interface in the KP.2 and KP.3 complexes is manifested in multiple side-chain conformations for H34 and K31 seen in the structural ensembles as well as the enhanced flexibility of the binding interface induced by L455S and F456L mutations. Overall, this analysis pointed to a central role of F456L as a potential regulator of epistatic couplings but also suggests a potential role of a structurally proximal Y453 site supporting the improved binding interfacial interactions in the KP.3 variant.

Our results also showed that Omicron variants can induce distinct dynamics and exploit epistatic interactions between sites R346, F486P, Q498, Q493 to modulate the RBD-ACE2 interface with increasing flexibility in antibody-binding residues [131]. The results demonstrated that L455, F456 and Q493 can serve as escape hotspots of resistance to class 1 antibodies. We suggested that epistatic couplings between these sites may not only help to restore ACE2-binding affinity but also represent a mechanism for amplifying immune response provided by individual mutations L455S, F456L and Q493E. These arguments are also consistent with evolutionary studies revealing strong epistasis between pre-existing substitutions in Omicron variants and antibody resistance mutations acquired during selection experiments, suggesting that epistasis can also lower the genetic barrier for antibody escape [132]. Based on the correspondence between the computational results and biochemical experiments we suggest that the primary role of BA.2.86, JN.1, KP.2 and KP.3 mutations may be to ensure a broad resistance against different classes of RBD antibodies, while several important mutations such as Q493E could confer the improved ACE2-binding affinity. Although the effect of immune evasion could be more variant-dependent and modulated through recruitment of mutational sites in various adaptable RBD regions, the currently dominating variants operate on a limited number of convergent escape hotspots. The results of our study provide further support to the mechanism in which epistatic interactions between convergent mutational sites may control proper balance between ACE2 binding and immune escape.

## 5. Conclusions

In this study, we combined AF2-based atomistic predictions of structures and conformational ensembles of the SARS-CoV-2 spike complexes with the host receptor ACE2 for the most recent dominant Omicron variants JN.1, KP.1, KP.2 and KP.3 to examine the mechanisms underlying the role of convergent evolution hotspots in balancing ACE2 binding and antibody evasion. Our multifaceted study explored quantitative aspects of structure, dynamics and energetics of the BA.2.86, JN.1 and KP.2/KP.3 variants, showing a robust agreement with a large body of experimental data on ACE2 binding and interactions with various classes of antibodies. The AF2-predicted conformational ensembles suggested the increased heterogeneity in the JN.1, KP.2 and especially KP.3 RBD variants which may potentially enable these variants to leverage a more mobile RBD structure to modulate and evade antibody neutralization. The results showed that the majority of mutational sites remain in similar positions in different conformations of the AF2 ensembles, but convergent mutational sites R346T, L455S, F456L and Q493E as well as in their interacting ACE2 sites K31, H34, E35 may exhibit a moderate level of flexibility and display concerted conformational rearrangements at the binding interface. Using conformational ensembles obtained from MD simulation studies of the RBD-ACE2 complexes for the BA.2.86, JN.1, KP.2 and KP.3 variants, we performed a systematic mutational scanning of the RBD residues in the RBD-ACE2 complexes. Our results suggested that F456L and Q493E may be only marginally deleterious and may reverse their effect on ACE2 binding in a different genetic background. Mutational profiling in the KP.3 background showed that the Q493E mutation becomes more favorable when combined together with KP.3 change L455S and especially F456L. The results of our study supported a recently proposed hypothesis that KP.2 and KP.3 lineages may have evolved to outcompete other Omicron subvariants by improving immune suppression while balancing binding affinity with ACE2 via the compensatory epistatic effect of L455S, F456L, Q493E and F486P mutations. Based on structural analysis of the conformational ensembles and MM-GBSA computations of the RBD-ACE2 complexes for JN.1, KP.2 and KP.3 variants, we argue that the conformational flexibility of the ACE2 interfacial sites that are afforded by L455S/F456L mutations can be properly exploited by Q493E, Y453 and F456L positions to enhance binding affinity. Overall, this analysis pointed to a central role of F456L as a potential regulator of epistatic couplings but also suggests a potential role of structurally proximal L452 and Y453 sites supporting the improved binding interfacial interactions in the KP.3 variant.

Structure-based mutational scanning of the RBD-binding interfaces with different classes of RBD antibodies characterized the role of specific mutations in eliciting broad resistance to neutralization against distinct epitope classes. The results demonstrated that JN.1, KP.2 and KP.3 variants harboring the L455SS, F456L and Q493E mutations can significantly impair the neutralizing activity of RBD class 1 monoclonal antibodies, also revealing that Y453, L455 and F456 emerged as major escape hotspots for these variants. These results are consistent with the experimental data showing that antibody evasion drives the convergent evolution of L455F/S and F456L, while the epistatic interactions mediated by F456L can facilitate the favorable contribution of Q493E to restore ACE2 binding. The results support the notion that evolution of Omicron variants may favor emergence of lineages with beneficial combinations of mutations involving mediators of epistatic couplings that control the balance of ACE2 affinity and immune evasion. Our study provided support to a mechanism in which convergent Omicron mutations can promote high transmissibility and antigenicity of the virus by controlling the interplay between the binding to the host receptor and robust immune evasion profile. This may potentially be a common strategy of Omicron evolution that would result in combinatorial exploration of convergent mutations to evade neutralizing antibodies and increase immune evasion potential while maintaining the acceptable ACE2 binding for productive viral transmission.

## Figures and Tables

**Table 1:** Mutational landscape of the Omicron variants.

Omicron Variant	Mutational Landscape
BA.1	A67, T95I, G339D, S371L, S373P, S375F, K417N, N440K, G446S, S477N, T478K, E484A, Q493R, G496S, Q498R, N501Y, Y505H, T547K, D614G, H655Y, N679K, P681H, N764K, D796Y, N856K, Q954H, N969K, L981F
BA.2	T19I, G142D, V213G, G339D, S371F, S373P, S375F, T376A, D405N, R408S, K417N, N440K, S477N, T478K, E484A, Q493R, Q498R, N501Y, Y505H, D614G, H655Y, N679K, P681H, N764K, D796Y, Q954H, N969K
XBB.1.5	T19I, V83A, G142D, Del144, H146Q, Q183E, V213E, G252V, G339H, R346T, L368I, S371F, S373P, S375F, T376A, D405N, R408S, K417N, N440K, V445P, G446S, N460K, S477N, T478K, E484A, F486P, F490S, R493Q reversal, Q498R, N501Y, Y505H, D614G, H655Y, N679K, P681H, N764K, D796Y, Q954H, N969K
BA.2.86	T19I, R21T, S50L, del69-70, V127F, delY144, F157S, R158G, delN211, L213I, L226F, H25N, A264D, I332V, D339H, K356T, R403K, V445H, G446, N450D, L452W, N460K, N481K, del V483, A484K, F486P, R493Q, E554K, A570V, P612S, I670V, H68R, D939F, P1143L
JN.1	T19I, R21T, S50L, del69-70,V127F, delY144, F157S, R158G, delN211, L213I, L226F, H25N, A264D, I332V, D339H, K356T, R403K, V445H, G446, N450D, L452W, **L455S**, N460K, N481K, del V483, A484K, F486P, R493Q, E554K, A570V, P612S, I670V, H68R, D939F, P1143L
KP.2	T19I, R21T, S50L, del69-70,V127F, delY144, F157S, R158G, delN211, L213I, L226F, H25N, A264D, I332V, D339H, **R346T**, K356T, R403K, V445H, G446, N450D, L452W, **L455S**, **F456L**, N460K, N481K, del V483, A484K, F486P, R493Q, E554K, A570V, P612S, I670V, H68R, D939F, **V1104L**, P1143L
KP.3	T19I, R21T, S50L, del69-70,V127F, delY144, F157S, R158G, delN211, L213I, L226F, H25N, A264D, I332V, D339H, K356T, R403K, V445H, G446, N450D, L452W, **L455S**, **F456L**, N460K, N481K, del V483, A484K, F486P, **Q493E**, E554K, A570V, P612S, I670V, H68R, D939F, **V1104L**, P1143L

**Table 2:** MM-GBSA binding energies for the BA.2.86, JN.1, KP.2 and KP.3 RBD-ACE2 complexes. The experimetal binding contants are from [48].

System	*E*_vdW_	*E*_elec_	*E_GB*	*E_SA*	Δ*G*_bind_ (kcal/mol)	Binding Constants (nM)
BA.2.86 RBD-ACE2	−83.57	−1438.64	1489.54	−10.23	−42.9	1.7 nM
BA.2.86 + L455S (JN.1) RBD-ACE2	−79.2	−438.12	1487.67	−9.66	−39.3	13.0 nM
BA.2.86 + F456L RBD-ACE2	−80.91	−1439.91	1489.21	−9.93	−41.54	12.0 nM
BA.2.86 + Q493E RBD-ACE2	−79.26	−1222.65	1269.94	−10.45	−42.42	Unknown
BA.2.86 + F456L/Q493E RBD-ACE2	−79.49	−1224.61	1270.23	−10.17	−44.04	Unknown
BA.2.86 + L455S/F456L (KP.2) RBD-ACE2	−77.40	−1440.2	1487.22	−9.31	−39.61	11.0 nM
BA.2.86 + L455S/Q493E RBD-ACE2	−77.2	−1222.66	1268.2	−9.97	−41.57	21.0 nM
BA.2.86 + L455S/F456L/Q493E (KP.3) RBD-ACE2	−77.96	−1226.20	1269.98	−9.60	−43.78	6.9 nM

## Data Availability

Data are fully contained within the article. Crystal structures were obtained and downloaded from the Protein Data Bank (http://www.rcsb.org) (accessed 25 April 2024). All simulations were performed using the NAMD 2.13 package that was obtained from https://www.ks.uiuc.edu/Development/Download/. All simulations were performed using the all-atom additive CHARMM36 protein force field that can be obtained from https://mackerell.umaryland.edu/ff_dev.shtml; The rendering of protein structures was performed with interactive visualization program UCSF ChimeraX package (https://www.cgl.ucsf.edu/chimerax/) and Pymol (https://pymol.org/). All the data obtained in this work are freely available at Zenodo: https://zenodo.org/records/12676345.

## Supplementary Materials

The following supporting information can be downloaded at: https://www.mdpi.com/article/10.3390/v16091458/s1, Figure S1: Structural alignment of the AF2-predicted RBD conformational ensemble using shallow MSA depth approach for the BA.2.86 RBD-ACE2 complex, JN.1 RBD-ACE2 complex, KP.2 RBD-ACE2 complex and KP.3 RBD-ACE2 complex. Figure S2: The distribution densities of pLDDT values and residue-based pLDDT profiles for the RBD of KP.2 and KP.3 RBD-ACE2 complexes. Figure S3: The distance fluctuation stability index profiles of the RBD residues obtained from MD simulations of the BA.2.86 RBD-ACE2 complex, JN.1 RBD-ACE2 complex, KP.2 RBD-ACE2 complex and KP.3 RBD-ACE2 complex. Figure S4: Structural overview of the binding interface for the S-RBD-ACE2 complexes of the Omicron XBB lineages. Figure S5: Ensemble-based mutational scanning of binding for the key RBD positions in backgrounds of the BA.2.86 variant based on MD simulations of the cryo-EM structure of the BA.2.86 RBD-ACE2 complex (pdb id 8QSQ). Figure S6: MM-GBSA binding free energy decomposition contribution of K31 and H34 ACE2 residues in BA.2.86, JN.1, BA.2.86 + F456L, BA.2.86 + Q493E, BA.2.86 + F456L/Q493E, KP.2, BA.2.86 + L455S/Q493E and KP.3 RBD-ACE2 complexes.
