# Supplementary material for: AlphaFold2 Modeling and Molecular Dynamics Simulations of the Conformational Ensembles for the SARS-CoV-2 Spike Omicron JN.1, KP.2 and KP.3 Variants: Mutational Profiling of Binding Energetics Reveals Epistatic Drivers of the ACE2 Affinity and Escape Hotspots of Antibody Resistance
Source: Viruses. 2024 Sep 13;16(9):1458. doi: 10.3390/v16091458 (PMC11437503; doi:10.3390/v16091458)
Supplement: Supplementary file 1 [file viruses-16-01458-s001.zip › viruses-3123330-Supplementary.pdf]

Supplementary Materials

AlphaFold2 Modeling and Molecular Dynamics Simulations of the Conformational Ensembles for the SARS-CoV-2 Spike Omicron JN.1, KP.2 and KP.3 Variants: Mutational Profiling of Binding Energetics Reveals Epistatic Drivers of the ACE2 Affinity and Escape Hotspots of Antibody Resistance

Nishank Raisinghani [1,2], Mohammed Alshahrani [1], Grace Gupta [1] and Gennady Verkhivker [1,3,*]

[1] Keck Center for Science and Engineering, Graduate Program in Computational and Data Sciences, Schmid College of Science and Technology, Chapman University, Orange, CA 92866, USA; rai.r.nick@gmail.com (N.R.), alshahrani@chapman.edu (M.A.), grgupta@chapman.edu (G.G.)

[2] Department of Structural Biology, Stanford University, Stanford, CA 94305, USA

[3] Department of Biomedical and Pharmaceutical Sciences, Chapman University School of Pharmacy, Irvine, CA 92618, USA

* Correspondence: verkhivk@chapman.edu; Tel.: +1-714-516-4586

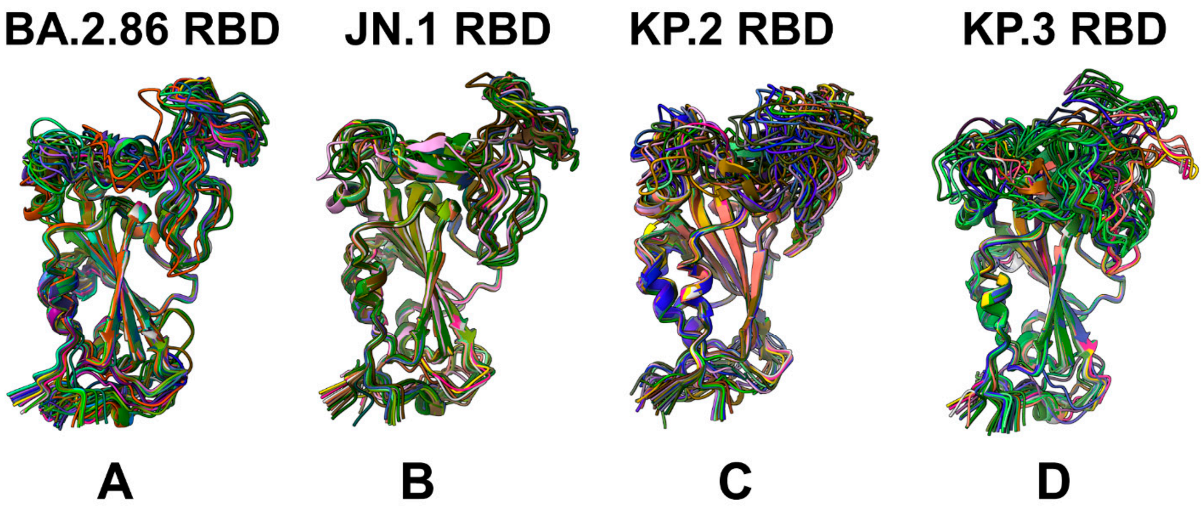

Figure S1. Structural alignment of the AF2-predicted RBD conformational ensemble using shallow MSA depth approach for the BA.2.86 RBD-ACE2 complex (A), JN.1 RBD-ACE2 complex (B), KP.2 RBD-ACE2 complex(C) and KP.3 RBD-ACE2 complex (D).

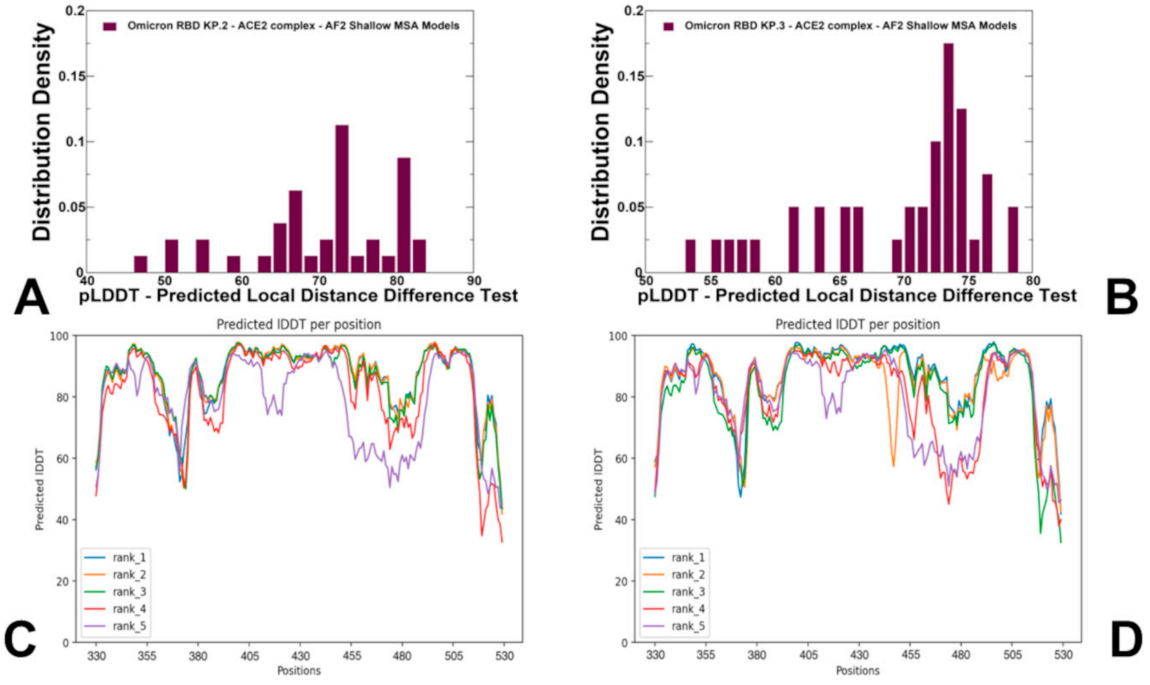

Figure S2. The distribution densities of pLDDT values and residue-based pLDDT profiles for the KP..2 and KP.3 RBD conformational ensemble obtained from AF2-MSA depth predictions. The density distribution of the pLDDT values for KP.2 (A) and KP.3 variants (B). The residue-based pLDDT profiles of the RBDN for KP..2 (C) and KP.3 (D) variants.

•

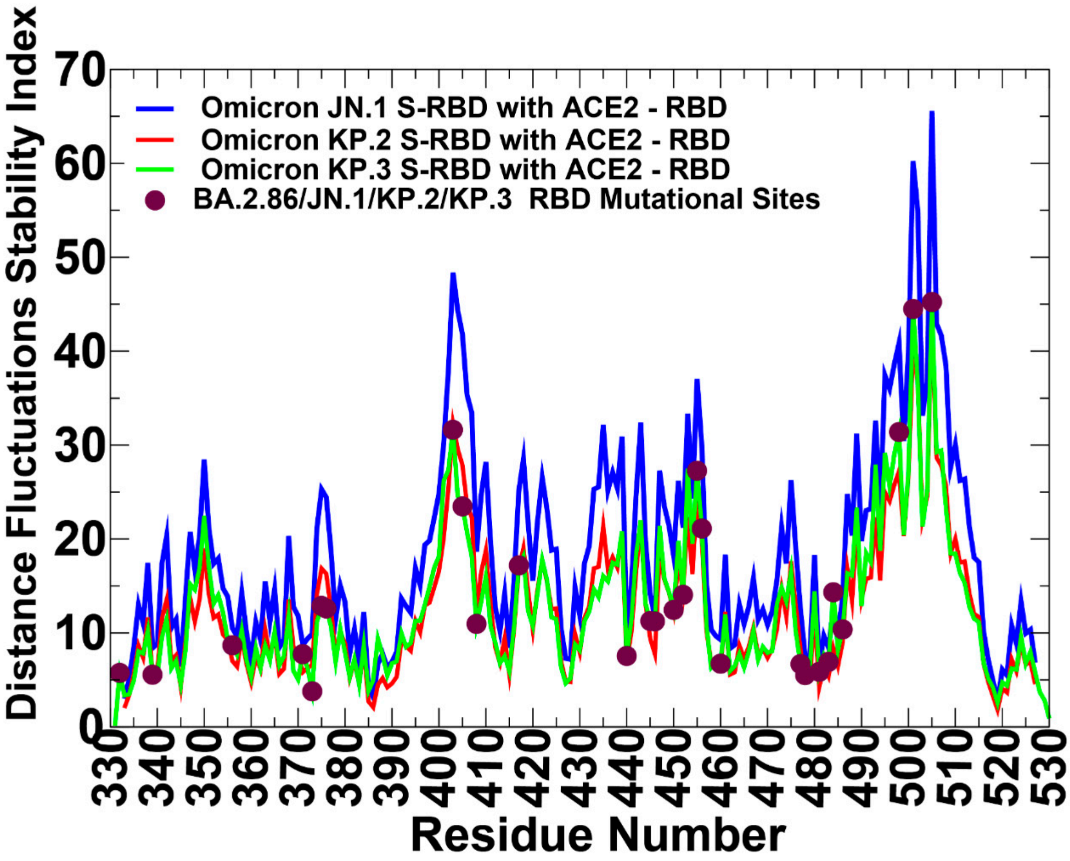

Figure S3. The distance fluctuations stability index profiles of the RBD residues obtained from MD simulations of the BA.2.86 RBD-ACE2 complex, JN.1 RBD-ACE2 complex, KP.2 RBD-ACE2 complex and KP.3 RBD-ACE2 complex. The profiles are shown for JN1 RBD (in blue lines) KP.2 RBD (in red lines), an KP3 RBD (in green lines) The positions of the BA.2.86/JN.1/KP.2/KP.3 mutations are highlighted in maroon colored filled circles.

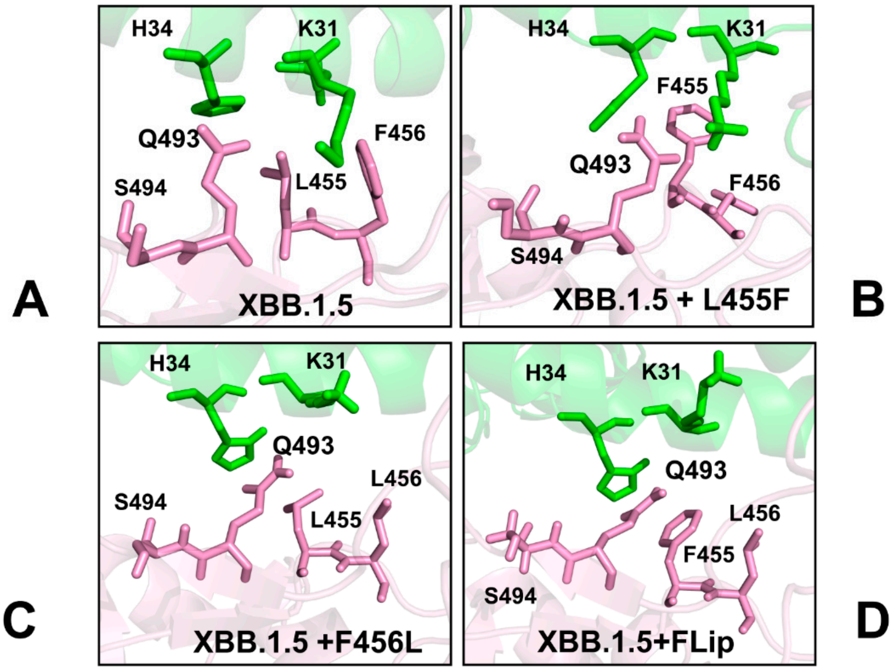

Figure S4. Structural overview of the binding interface for the S-RBD-ACE2 complexes of the Omicron XBB lineages. (A) A closeup of the binding interface residues Q493, L455 and F456 in the cryo-EM structure of the XBB.1.5 RBD-ACE2 complex, pdb id 8WRL. The RBD residues are in cyan sticks, the ACE2 residues H3 and K31 are in green sticks. (B) A closeup of the binding interface residues Q493, L455F and F456 in the AF2-predicted best model of the XBB.1.5+L455F complex with ACE2. (C) A closeup of the binding interface residues Q493, L455 and F456L in the cryo-EM structure of the XBB.1.5+F456L complex with ACE2, pdb id 8WTD (C) A closeup of the binding interface residues Q493, L455F and F456L in the cryo-EM structure of the XBB.1.5+L455F/F456L FLip complex with ACE2, pdb id 8WRH. The binding interface residues are shown in pink sticks for RBD sites and green sticks for ACE2 sites. The RBD interface residues Q493, L455F and F456L undergo rearrangements in the XBB.1.5+FLip complex.

c

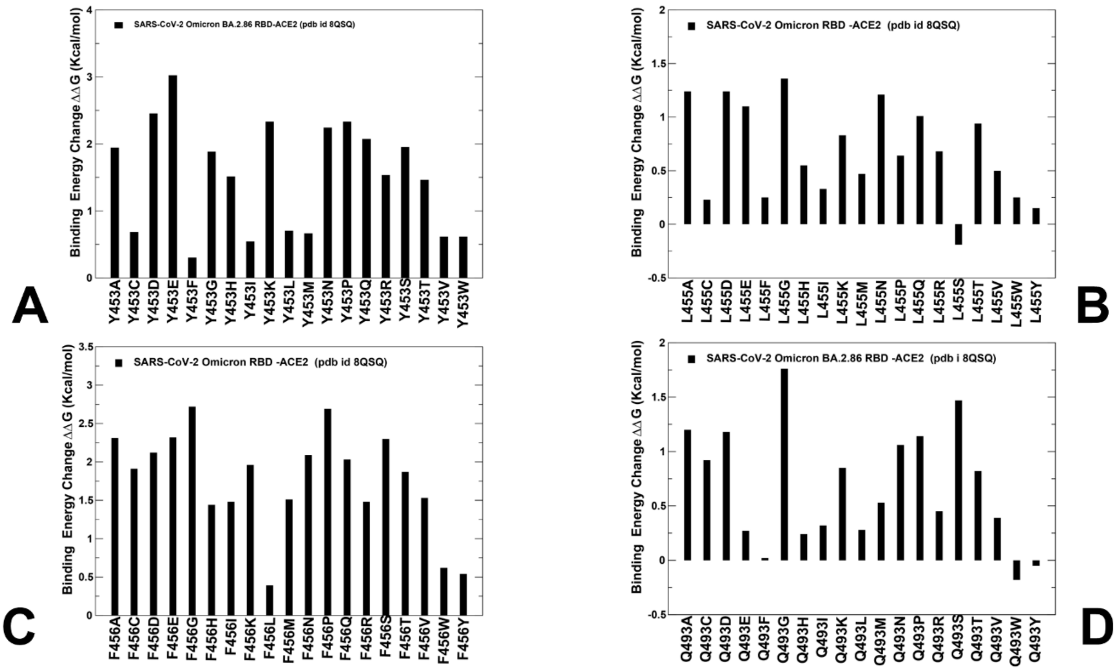

Figure S5. Ensemble-based mutational scanning of binding for the key RBD positions in backgrounds of the BA.2.86 variant based on MD simulations of the cryo-EM structure of the BA.2.86 RBD-ACE2 complex (pdb id 8QSQ). The profiles of computed binding free energy changes ΔΔG upon 19 single substitutions for Y453 residue in BA.2.86 (A), L455 in BA.2.86 (B), F456 in BA.2.86 (C), and Q493 in BA.2.86 (D. The binding free energy changes are computed using conformational ensembles obtained from MD simulations. The binding free energy changes are shown in black-colored filled bars. The positive binding free energy values ΔΔG correspond to destabilizing changes and negative binding free energy changes are associated with stabilizing changes.

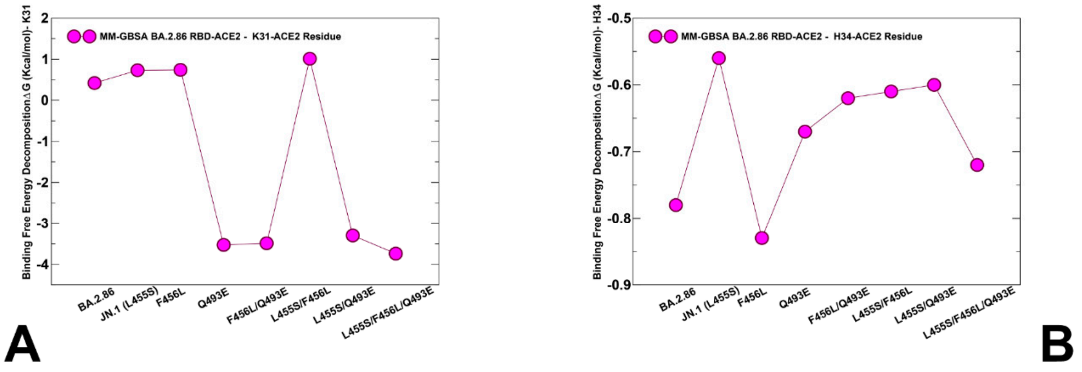

Figure S6. MM-GBSA binding free energy decomposition contribution of K31 and H34 ACE2 residues in BA.2.86, JN.1, BA.2.86+F456L, BA.2.86+Q493E, BA.2.86+F456L/Q493E, KP.2, BA.2.86+L455S/Q493E and KP.3 (RBD-ACE2 complexes. (A) The MM-GBSA decomposition contribution of the binding energies for K31-ACE2 residue. (B) The MM-GBSA decomposition contribution of the binding energies for H34-ACE2 residue.
